# From MS/MS library implementation to molecular networks: Exploring oxylipin diversity with NEO-MSMS

**DOI:** 10.1038/s41597-024-03034-4

**Published:** 2024-02-13

**Authors:** Anis Elloumi, Lindsay Mas-Normand, Jamie Bride, Guillaume Reversat, Valérie Bultel-Poncé, Alexandre Guy, Camille Oger, Marie Demion, Jean-Yves Le Guennec, Thierry Durand, Claire Vigor, Ángel Sánchez-Illana, Jean-Marie Galano

**Affiliations:** 1 Institut des Biomolécules Max Mousseron (IBMM), UMR 5247-CNRS, 34293 Montpellier, France; 2 PhyMedExp, Université de Montpellier, Inserm U1046, UMR CNRS 9412, Montpellier, France; 3 Department of Analytical Chemistry, University of Valencia, Dr. Moliner 50, 46100 Burjassot, Spain

**Keywords:** Mass spectrometry, Scientific data, Bioanalytical chemistry

## Abstract

Oxylipins, small polar molecules derived from the peroxidation of polyunsaturated fatty acids (PUFAs), serve as biomarkers for many diseases and play crucial roles in human physiology and inflammation. Despite their significance, many non-enzymatic oxygenated metabolites of PUFAs (NEO-PUFAs) remain poorly reported, resulting in a lack of public datasets of experimental data and limiting their dereplication in further studies. To overcome this limitation, we constructed a high-resolution tandem mass spectrometry (MS/MS) dataset comprising pure NEO-PUFAs (both commercial and self-synthesized) and *in vitro* free radical-induced oxidation of diverse PUFAs. By employing molecular networking techniques with this dataset and the existent ones in public repositories, we successfully mapped a wide range of NEO-PUFAs, expanding the strategies for annotating oxylipins, and NEO-PUFAs and offering a novel workflow for profiling these molecules in biological samples.

## Background & Summary

Lipid peroxidation was mechanistically revealed in the 1940s thanks to the scientists at the British Rubber Products Association, and only then, the role of free radicals derived from molecular oxygen started to make sense leading to the understanding of the autooxidation phenomena^1^. Soon enough, the discovery of arachidonic acid (AA) cascade (enzyme-catalyzed peroxidation) led to the discovery of bioactive eicosanoids, and oxylipins produced by other polyunsaturated fatty acids (PUFA) than AA^2^. Finally, non-enzymatic oxygenated metabolites of polyunsaturated fatty acids (NEO-PUFAs) generated by triplet or singlet oxygen-dependent reactions were originally identified as mere product of oxidative stress (hence potential biomarkers), however several lines of evidence have highlighted their role as lipid mediators such as the isoprostanoids^3^.

Early methods for detection and quantification of oxylipins and NEO-PUFAs in samples relied on thin-layer chromatography (TLC), gas chromatography (GC), and mass spectrometry (MS). Over the past three decades, advances in hyphenated techniques such as liquid chromatography coupled to tandem mass spectrometry (LC-MS/MS) along with high resolution MS and novel data analysis approaches have allowed the comprehensive analysis of the various modifications of lipids by enzymatic and non-enzymatic reactions, forming higher levels of structural complexity, known as the epilipidome^4^.

One of the challenges in epilipidomics is the identification of the modified lipids, especially so if they were already discovered, i.e., the dereplication of oxidized lipids^5^.

While efforts are being currently underway for the practical identification of oxylipins esterified to complex lipids^6^, a recent work by Watrous *et al*.^7^ reported the dereplication of free oxylipins by molecular networking^8,9^ using a high resolution mass spectrometry (HRMS) directed non-targeted approach, thanks to reference tandem mass spectra of 234 of commercially available oxylipins, and a few NEO-PUFAs. Interestingly, they reported the discovery of more than 500 novel oxylipins (called “putative” in the text). This number should have raised more interest to the community, as it is believed that very little gap remains for novel oxylipin structures. We initially hypothesized that the lack of MS/MS libraries for NEO-PUFAs could partially explain the “putative” oxylipins number. Although a large amount of knowledge over the last 25 years has been accumulated concerning the relevant NEO-PUFAs detected by LC-MS/MS, it appears impossible to gather full spectra MS/MS data from either the reports of their discovery or from their synthetic preparation. Knowing that targeted MRM (multiple reaction monitoring) on low resolution mass spectrometry is preferred for quantification of NEO-PUFAs, full MS/MS data repositories could seem irrelevant at the time of their discovery. However very few of those discovered NEO-PUFAs were made available making todays dereplication not feasible. This problematic is recurrent in the field, and a recent investigation proved the importance of data repositories^10^. Recent studies toward the development and optimization of analytical strategies based on HRMS showed great promises, and improvement of spectrometer sensitivity will surely keep on improving^11,12^ allowing mainstream use.

As part of our continuing interest in NEO-PUFA chemistry, we developed a streamlined molecular networking dereplication pipeline based on the implementation of a MS/MS library that we named non-enzymatic oxylipins MS/MS library (NEO-MSMS). It contents an in-house collections of synthesized and unique NEO-PUFA standards, commercially available enzymatic oxylipins, and a curated MS/MS data collection of commercially available oxylipins from Watrous *et al*. study^7^. Moreover, in vitro non-enzymatic oxidation of the four main PUFA (AA, eicosapentaenoic acid [EPA], docosahexaenoic acid [DHA] and α-Linolenic acid [ALA]) lead to the creation of a vast MS/MS dataset of NEO-PUFAs which was sought to extend the coverage signature of oxylipins.

**Fig. 1:**
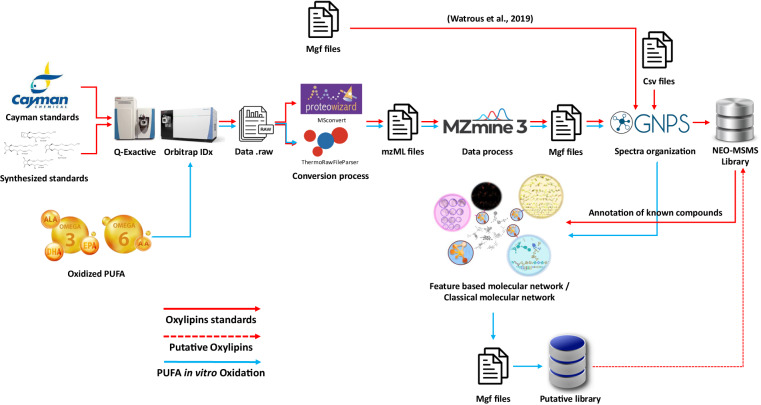
Oxylipins/NEO-PUFAs library implementation workflow (red path) and application in a molecular networking based dereplication workflow (blue path).

This data descriptor presents the deposition of all this data in Zenodo and MASSIVE repositories along with its subsequent technical validation. Additionally, we also present the inclusion of the NEO-MSMS as a compatible GNPS library augmented with putatively annotated MS/MS oxylipin spectra extracted from the in vitro oxidation (Fig. 1).

We believe this information could set the ground for understanding the importance of building a MS/MS library dedicated to oxygenated PUFA metabolites, and we hope natural product researchers, organic chemists and lipidomists in the field to share their collections, expand and use NEO-MSMS.

## Methods

### Standards and reagents

All solvents and reagents employed were ≥98% purity unless otherwise indicated, including cyclohexane, acetonitrile, methanol, ethanol absolute, ammonia solution 30% (w/w), and water, all sourced from Fisher Chemicals (Waltham, MA, USA). Hexane, ethyl acetate, potassium hydroxide, sodium hydroxide, sodium chloride, potassium chloride, calcium chloride, magnesium chloride, monosodium phosphate, disodium phosphate, glucose, trimethyl phosphite, acetic acid, and formic acid were obtained from Merck (Darmstadt, Germany). The free radical initiator 2, 2′-azobis(4-methoxy-2, 4-dimethylvaleronitrile) (V70) was procured from FUJIFILM Wako Chemicals (Tokyo, Japan) and N-methyl benzohydroxamic acid (NMBHA) was prepared in our laboratory following the methodology outlined in a prior publication^13^.

Tyrode’s solution was prepared weighting and dissolving in water the appropriated amounts of the following salts to obtain the concentration of NaCl 8 g/L, KCl 0.2 g/L, CaCl_2_ 0.2 g/L, MgCl_2_ 0.1 g/L, NaH_2_PO_4_ 0.05 g/L, NaHCO_3_ 1 g/L, and glucose 1 g/L. The pH was adjusted with 1 M solution NaOH to 7.4.

The PUFAs used in the *in vitro* oxidation were AA and EPA from Merck (Darmstadt, Germany), DHA from Santa Cruz Biotechnology (Dallas, TX, USA), and ALA from BLDpharma (Shanghai, China). The PUFA esters employed were DHA ethyl ester, AA methyl ester, and EPA methyl ester from Merck (Darmstadt, Germany) and ALA methyl ester purchased from Fisher Chemicals (Waltham, MA, USA).

Regarding oxylipin standards, individual stock solutions at 1 µmol/L of 131 oxylipins (purity >95%) were prepared dissolving the appropriate amounts in H_2_O:CH_3_CN mixture (i.e. 20% CH_3_CN). This includes 72 commercially available enzymatic oxylipins and NEO-PUFAs purchased at Cayman Chemical (Ann Arbor, MI) as individual standard or premixed solution and 59 NEO-PUFAs obtained by total synthesis in our laboratory following previously developed procedures and purity-checked by NMR^14–21^.

As internal standards (IS); the same mixture of oxylipins commonly employed in our laboratory for quantitative methods published elsewhere^22,23^ weighting the appropriate amounts of compounds and dissolving it in H_2_O (20% CH_3_CN) to obtain a concentration of 3.25 mg/L each. This includes non-natural odd number analogues of oxylipins not produced by mammal cellular process C19-16-F_1t_-PhytoP and C21-15-F_2t_-IsoP, and an isotopically labelled D4-10-*epi*-10-F_4t_-NeuroP.

### *In vitro* radical-induced oxidation of PUFAs

To encompass the broad spectrum of NEO-PUFAs produced via triplet oxygen-dependent reactions from PUFAs, various oxidation protocols were applied to DHA, ALA, AA, and EPA acids and esters. The objective was to generate precise samples for encompassing the chemical signature of NEO-PUFAs (oxPUFA data in the text).

#### Oxidation of PUFAs with H_2_O_2_

Approximately 5 mg of free fatty acid (≈17 µmol) were dissolved in 100 µL of ethanol. Subsequently, it was mixed with a 1.8 mL Tyrode’s solution and 100 µL H_2_O_2_ solution (120 mM in water) to reach a final volume of 2 mL and approximate concentrations of 8.5 mM and 6 mM for PUFA and H_2_O_2_, respectively (i.e., 1.4 PUFA/H_2_O_2_ molar ratio). The oxidation proceeded for 30 minutes, then the reaction mixture was divided in two (1 mL each) aliquots. In one aliquot, 0.9 mL of 1 M KOH in water was added and incubated for 30 minutes at 40 °C with mid rotation in tube rotator. After this KOH saponification, 100 µL of formic acid was added to obtain an acidic pH of 4. In the other aliquot, only 1 mL of acidified water with formic acid (pH = 4) was added.

Of note: this alkaline hydrolysis step was included as a control experiment since best practice for NEO-PUFAs profiling requires such step in the extraction procedure of plasma/tissues (to the opposite of oxylipins), and that it was reported to transform or degrade a few NEO-PUFA/oxylipin structures^24,25^. Other oxylipin metabolites possessing 3, 5-disubstituted 1, 2-dioxolane units (endoperoxides) are also known to rearrange under basic conditions via the Kornblum-DeLaMare reaction onto ketohydroxyl derivatives (i.e; PGG_2_ into PGE_2_), however the fate of endoperoxide metabolites (derived from initial 5-exo-trig cyclization followed by oxygenation of peroxyl radical precursor) remains uncertain. On model studies^26,27^, linear 1, 2-dioxolanes do not rearrange under our alkaline conditions (see Supplementary Information Figure S1). Other endoperoxide derivatives (like PGGs) were not investigated.

Each aliquot (i.e., saponified and non-saponified) was purified by automated solid phase extraction (SPE) process employing Extrahera system from Biotage (Uppsala, Sweden) and Oasis Max cartridge as stationary phase (60 mg, 30 µm particle size) from Waters (Mildford, MA, USA). The SPE procedure was optimized for the extraction of oxylipins in our previous works;^22,23,28^ briefly, after the conditioning and sample loading, the cartridge was washed with 2 mL each of 2% NH_3_ solution, MeOH/formic acid (20 mM, 30:70), pure hexane, and hexane/ethyl acetate (70:30). Subsequently, elution is carried out using 1.5 mL of hexane/EtOH/acetic acid (70:29.4:0.6). Finally, each aliquot elution was divided in two again (750 µL each) and 50 µL of P(OMe)_3_ was added in one of the aliquots and incubated 30 min at room temperature in order to reduce hydroperoxide derivatives and bicyclic endoperoxides, keeping intact linear endoperoxides (1, 2-dioxolanes)^29^. The robustness of the linear endoperoxides was demonstrated through reduction tests on an isolated compound (Supplementary Information Figure S1).

The four final samples of oxidized PUFA (oxPUFA) per every single PUFA investigated (i.e., saponified/non-saponified with/without P(OMe)_3_ reduction) were evaporated with CentriVap Vacuum Concentrator (Labconco, Kansas, MO, USA) and resuspended with 970 mL of H_2_O (20% CH_3_CN) plus 30 µL of IS mixture.

#### Oxidation of PUFA ethyl esters with NMBHA

Adapted from refs. ^30,31^; approximately 50 mg (≈170 µmol) of esterified fatty acid was diluted in 2 mL of anhydrous CH_3_CN, mixed with 7 mg of V70 (≈23 µmol), followed by addition of 25 mg of N-methyl benzohydroxamic acid (NMBHA) (≈170 µmol), and incubated for 24 hours opened to room air (i.e. 21% O_2_) at 37 °C in sand bath. The oxidation product was purified to eliminate NMBHA, the unreacted PUFA and other by-products with Flash-LC employing a Reveleris X2 system from Buchi (Eastern Switzerland) and a puriflash 30SI-JP/12 g 30 µm column from Interchim (Montluçon, France). Solvent was eliminated with rotavap, resuspended in 1 mL of cyclohexane with a few drops of ethyl acetate until soluble material could be injected into the Flash-LC system. The separation gradient was cyclohexane (0.1% acetic acid) (A) and ethyl acetate (0.1% acetic acid) (B) as eluents, and the flow rate was set at 30 mL/min, (25 mL per tube). The proportion of B was gradually increased from 0% to 10% over 6.2 minutes, then to 23% over 2.3 minutes, then maintained isocratic for 4.7 minutes and increased to 100% over 0.7 minutes which was held 5 minutes for a total run time of 18.9 minutes. The elution of the unreacted PUFA, oxPUFA, and the NMBHA were controlled by TLC and only the fractions of oxPUFA were finally collected. It should be noted that the amount of NMBHA employed could be very problematic if it was injected concentrated into the LC-MS system so this cleaning step is mandatory. The oxPUFA fractions were concentrated with rotavap collecting approximately 25 mg of oxPUFA of which 5 mg were diluted in 2 mL of water to reach the same concentration as in the H_2_O_2_ oxidation. Then, it was proceeded to saponification, SPE, P(OMe)_3_ reduction and IS dilution such as in the H_2_O_2_ oxidation.

#### Oxidation of ethyl esters of PUFAs without NMBHA

The last oxidation was made accordingly to the previous one but without the addition of NMBHA. Additionally, during Flash-LC purification two fractions of oxPUFA were collected, i.e., the ones before and after the hypothetical elution of NMHBA. This was done to access to the oxylipins coeluting with NMBHA and lost in the NMBHA oxidation.

### *In vitro* radical-induced oxidation or rearrangement of oxylipins

Microscaling the previously reported leukotriene-like formation by oxidation of 15-HETE described by Rector *et al*.^32^ and retrieve the partially reported information (MS/MS data) could be an answer to redeem annotation of oxylipins uncovered in past studies. Furthermore, solvent-assisted rearrangement of PGH_1_ and PGH_2_ was microscaled with the hope of acquiring the MS/MS data of the main produced metabolites, i.e., the 15-levuglandins LGE_1_ and LGE_2_^33^.

#### Microscaled V70 oxidation of commercially oxylipins

A solution of 30 µL of V70 (0.15 mol/L) in CH_3_CN was added to 50 µg of 15-HETE redisposed in chromatographic glass vials with 250 µL insert with conical bottom and quickly rotated with small angle using a Bio PTR-35 360° Multi-Functional Tube Rotator from Grant Instruments, Royston, UK) at 40 °C for 24 h in an oven. The agitation conditions were the following: 100 rpm orbital, a reciprocal rotation of 11° turning angle for 6 seconds followed by a 2° vibration for 1 second. The solution is then evaporated with vacuum concentrator and diluted in 100 µL of mobile phase before LC-MS injection.

#### PGH rearrangement

Commercially available standards PGH_2_ (50 µg) and PGH_1_ (25 µg) were placed in chromatographic glass vials with 250 µL insert with conical bottom, dissolved in 30 µL of DMSO^33–35^, and mechanically agitated 1 hour at 37 °C using the tube rotator with the same parameters of microscaled V70 oxidation. Finally, 270 µL of mobile phase were added immediately before the LC-MS injection.

### Blood serum and microalgae samples

We used four samples of oxylipin extractions from human blood serum and three from microalgal biomass to demonstrate the practical application of NEO-MSMS and oxPUFA data with real samples. Serum samples had been processed for the quantitative profiling of oxylipins/NEO-PUFAs following our extraction protocol^22,23,28^ (including the KOH saponification and the SPE like the oxPUFA samples described above) and the extracts stored at – 80 °C. Blood samples of healthy donors were obtained from EFS (Etablissement Français du Sang, Montpellier). All procedures in use by the EFS are defined by the law and follow institutional guidelines.

Similarly, the microalgae samples had been extracted in the framework of a previous study including the sample grinding and multiple centrifugation and purification steps^36,37^.

### Data acquisition

Three different methods were developed with two different LC-MS/MS platforms. On the one hand, a Vanquish UHPLC system coupled to Q-Exactive Focus orbitrap mass spectrometer from Thermo Fisher Scientific (Waltham, MA, USA) was used for the LC-MS/MS spectra acquisition of standards (method 1). On the other hand, a Vanquish UHPLC system coupled to Orbitrap ID-X Tribrid mass spectrometer, also from Thermo Fisher Scientific, was used for the Flow injection analysis-MS/MS (FIA-MS/MS) spectra acquisition of standards (method 2); and for the LC-MS/MS spectra acquisition of standards, *in-vitro* oxidation, and proof-of-concept analysis of samples (method 3). The experimental conditions for each method are summarized in Table 1.

**Table 1:** Summary of the instrumental parameters of the methods employed in this work.

**Method 1**.	
Types of samples analyzed	Individual standards and standard mixtures
LC-MS/MS system	Vanquish UHPLC system coupled to Q-Exactive Focus
LC column	Kinetex EVO C18 (100 × 2.1 mm column 1.7 µm) from Phenomenex (Torrance, CA, USA)
Injection volume	5 µL
LC conditions	0.3 mL/min binary gradient using H_2_O (0.1% HCOOH) (i.e. channel A) and CH_3_CN:CH_3_OH 8:2, (0.1% HCOOH) (i.e. channel B). Solvent B was increased from 10% to 20% from 0 to 1.00 minute, 20% to 55% from 1.00 to 12.00 minutes, 55% to 61% from 12.00 to 12.30 minutes, 61% to 81% from 12.30 to 18.5 min, 81% to 99% from 18.30 to 19.00 minutes, 99% of B from 19.00 to 21.00 minutes, 99% to 10% B from 21.00 to 22.00 minutes and 10% B from 22.00 to 24.00 minutes.
Interface conditions	H-ESI in negative mode, spray voltage 3.5 kV, ion transfer tube temp 265 °C, sheath gas flow rate 40 L/min, auxiliary gas flow rate 15 L/min, spare gas 2 L/min, probe heater temperature 350 °C.
DDA parameters	Survey scan: resolution 35k, scan range 200–450 m/z, AGC target 1E6, profile mode
	MS/MS scans: resolution 17k, isolation window 1 Da, CID collision energy 30 eV, AGC target 2E5, 3 top intense MS1 ions fragmented, dynamic exclusion 0.3 s, exclude isotopes, profile mode.
	Inclusion list used with theoretical exact mass of oxylipins.
**Method 2**.	
Types of samples analyzed	Individual standards
LC-MS/MS system	Vanquish UHPLC system coupled to Orbitrap ID-X Tribrid
Injection volume	10 µL
FIA conditions	1 minute 0.5 mL/min isocratic at 50% H_2_O (0.1% HCOOH):50% CH_3_CN (0.1% HCOOH)
Interface conditions	H-ESI in negative mode, spray voltage 2.5 kV, sheath gas flow rate 60 L/min, auxiliary gas flow rate 15 L/min, sweep gas 2 L/min, ion transfer tube temp 350 °C, vaporizer temperature 400 °C.
DDA parameters	Survey scan: resolution 60k, scan range 100–600, AGC target 4E5, profile monde, EASY-IC internal calibration
	Intensity threshold filter: min. intensity 2E4,
	MS/MS scans: resolution 30k, isolation window 1 Da, stepped HCD collision energy 20–40–60%, AGC target 5E4, 0.5 s cycle time, centroid mode, dynamic exclusion time 2 s, EASY-IC internal calibration.
**Method 3**.	
Types of samples analyzed	Standard mixtures, *in vitro* oxidation, and test samples
LC-MS/MS system	Vanquish UHPLC system coupled to Orbitrap ID-X Tribrid
LC column	Kinetex C18 (100 × 2.1 mm, 1.7 µm) from Phenomenex (Torrance, CA, USA)
Injection volume	5 µL
LC conditions	H_2_O (0.1% HCOOH) (i.e. channel A) and CH_3_CN (0.1% HCOOH) (i.e. channel B) binary gradient at 0.5 m L/min. The gradient was as follows: 10% to 20% of B from 0 to 0.63 minute, 20% to 55% from 0.63 to 7.5 minutes, 55% to 57% from 7.5 to 7.69 minutes, 57% to 61% from 7.69 to 10.69 minutes, 61% to 81% from 10.69 to 14.75 minutes, 81% to 99% from 14.75 to 14.8 minutes 99% B from 14.80 to 15.80 minutes, 99% to 10% from 15.8 to 16 minutes and 10% of B from 16 to 17 minutes with a re-equilibration of 1 minute at 10% of B.
Interface conditions	H-ESI in negative mode, spray voltage 2.5 kV, sheath gas flow rate 60 L/min, auxiliary gas flow rate 15 L/min, sweep gas 2 L/min, ion transfer tube temp 350 °C, vaporizer temperature 400 °C.
DDA parameters	Survey scan: resolution 60k, scan range 100–600, AGC target 4E5, profile mode, EASY-IC internal calibration
	Intensity threshold filter: min. intensity 2E4,
	MS/MS scans: resolution 30k, isolation window 1 Da, stepped HCD collision energy 20–40-60%, AGC target 5E4, 0.5 s cycle time, centroid mode, dynamic exclusion time 2 s, EASY-IC internal calibration.
	Inclusion list used with theoretical exact mass of [M-H] ions of oxylipins*.

*Note: the inclusion lists used are available in Supplementary Table S3 of this data descriptor.

#### MS/MS spectra of standards

A total of 54 and 57 individual oxylipin standards were injected with the methods 1 and 2, respectively, and 28 with both methods. Furthermore, 2 and 6 mixtures of oxylipin standards were injected with the methods 1 and 3, respectively. Altogether, it resulted in 119 files in.raw format. The details of the standards and the composition of the mixtures are available in Supplementary Tables S1, S2.

#### Analysis of oxPUFA and oxidized oxylipins by LC-MS/MS data dependent acquisition (DDA)

All samples were randomly injected with the method 3 in two batches. Analysis of the first batch consisted of the 40 oxPUFA samples, a pooled quality control sample (QC), which was prepared mixing 5 µL of each oxPUFA sample, and a blank sample both injected each 10^th^ samples. Different DDA inclusion lists were made for each PUFA oxidation calculating the theoretical molecular formulae after adding different levels of oxidation following the known mechanisms of PUFA oxidation. The inclusion lists are available in Supplementary Table S3. The second batch contains the microscaled oxidized 15-HETE, and DMSO-rearranged PGH_1_, and PGH_2._

#### Analysis of blood serum and microalgae samples by LC-MS/MS DDA

The processed blood serum and microalgae samples were injected into the LC-MS/MS system following the method 3. A combine inclusion list of the four different inclusion lists (per PUFA) was used.

### NEO-MSMS library building

The analysis of standards yielded 119 files in.raw, of which 56 were from method 1 and 63 from methods 2 and 3. The files were converted to centroided.mzML format using MSconvert^38^ (version 3.0.23056) for the method 1 files, while ThermoRawFileParser^39^ version 1.4.2 was used for the Orbitrap IDx files (i.e. methods 2 and 3). This last conversion allowed to remove the fluoranthene calibration masses from the MS1 and MS2 spectra. Subsequently, the MS/MS spectrum for each standard was manually selected using MZmine3 MS spectra visualizer tool (version 3.7.0)^40^, focusing on the peak apex identified through the extracted ion chromatogram (EIC) with a 3 ppm mass error relative to the theoretical [M-H] precursor. When a MS/MS was extracted from a mixture of standards, the identity of each isobaric feature was confirmed by the relative retention time and specific MS/MS fragments published in our previous works^3^ or in the mixture datasheet for commercial mixtures. Then, the MS/MS spectra were converted to.mgf format resulting in 195 different spectra of which 131 are unique compounds. This.mgf files were submitted to GNPS libraries^8,9,41^ employing the ‘Batch Upload of Annotated Spectra’ workflow (https://ccms-ucsd.github.io/GNPSDocumentation/batchupload/). Additionally, another submission was done employing 217 MS/MS.mgf files of commercial standards obtained from Watrous *et al*. study focused on enzymatic oxylipins^7^. This building procedure was summarized in Fig. 1.

### Classical Molecular network of standards

Every individual MS/MS spectrum obtained according to the library building section were subjected to classical molecular networking^8^ using the GNPS platform (https://gnps.ucsd.edu/). The parent mass tolerance and MS/MS fragment ion tolerance were set at 0.02 Da for the analysis. The data were not clustered using MS-Cluster. The resulting network was filtered based on edges, ensuring a cosine score above 0.71 and a minimum of 5 matched peaks. Additionally, edges between two nodes were retained in the network only if each node appeared in the respective top 10 most similar nodes of the other node. To allow for an unlimited number of nodes in a single network, the maximum size of nodes was set to 0 in the connected network.

### Feature-based molecular networking of oxPUFAs

The LC-MS/MS data files associated with oxPUFAs were converted from the.raw data format to centroided.mzML files using the ThermoRawFileParser version 1.4.2^39^. Subsequently, the.mzML file was processed further using MZmine3 (version 3.7.0)^40^. Mass detections were conducted with a noise level threshold of 2E4 in MS1 and 2E3 in MS/MS. The ADAP chromatogram builder utilized a minimum group size of 4 scans, a group intensity threshold of 5E3, a minimum highest intensity of 1E4, and an m/z tolerance of 3 ppm. Chromatogram deconvolution employed the Local Minimum Search algorithm with the following settings: chromatographic threshold = 50%, search minimum in RT range (min) = 0.03, minimum relative height = 1%, minimum absolute height = 2E4, min ratio of peak top/edge = 1, peak duration range (min) = 0.00–1 and minimum scans (data points) = 4. The extracted features of the same PUFA were aligned together using a m/z tolerance of 3 ppm, and retention time tolerance of 0.03 min. The peak list was filtered to retain only features with MS/MS features. All features present in the blank samples have been removed, unless the intensity (peak height) of the feature is 300% more intense than that present in the blank. The alignment was checked with the ISs features which were picked and aligned correctly across samples. The QC data was not used in this alignment approach but provided as a part of this data descriptor for further studies more focused on the quality assurance of semi quantification. All even-mass features have been removed to simplify the studied molecular network. Gap filling is employed to reduce false missing values, using an intensity tolerance of 20%, an m/z tolerance of 3 ppm, and a minimum of 5 scans. Also, an additional feature table was obtained by aligning the feature tables from each PUFA using the same parameters stated before (i.e., all PUFA alignment). The resulting.mgf and.csv files were exported using the built-in “Export/Submit to GNPS/FBMN” module in MZmine3 using the merge MS/MS approach. It was employed with specific parameters to enhance the analysis. Spectra were selected for merging across samples, and the M/Z merge mode utilized a weighted average with the removal of outliers. Intensity merge mode operated by summing intensities. The criteria for merging included an expected mass deviation of 0.001 m/z or 5 ppm, ensuring precision in the spectral combination. A cosine threshold of 70% was set to evaluate the similarity between spectra, and a signal count threshold of 20% was implemented to filter out low-intensity signals. The isolation window offset was set at 0 m/z, and the window width was established at 3 m/z. Finally, the molecular networks were created using the online FBMN workflow^42^ (version release_28.2) at GNPS platform (https://gnps.ucsd.edu/) with a parent mass tolerance of 0.02 Da and an MS/MS fragment ion tolerance of 0.02 Da. Edges in the network were filtered to have a cosine score above 0.6 and more than 5 matched peaks. Additionally, edges between two nodes were retained only if each node appeared in the respective top 15 most similar nodes of the other and a maximum connected component size of 50.

The spectra in the network were compared against GNPS spectral libraries and our 3 libraries built in this work. Matches between network spectra and library spectra were required to have a cosine score above 0.6 and at least 5 matched peaks. The molecular networking data were analyzed and visualized using Cytoscape (ver. 3.10.0) and was used for the dereplication of new putative NEO-PUFAs studying the fragmentation patterns as described in the Technical Validation section. The construction of the pie charts that will be visualized on the feature based molecular network (FBMN) is based on the peak heights of the corresponding feature in each sample.

### Data analysis of oxidized and rearranged oxylipins

The identification of putative oxylipins from the 15-HETE oxidation and PGH rearrangement experiments was performed by extracting the EICs of specific parent masses on MZmine3 (version 3.7.0) with 3 ppm tolerance and extracting the MS/MS spectra of the integrated features at peak apex. Subsequently, the identification is based on the study of MS/MS data based on known oxylipin fragmentation mechanisms.

### Molecular networking of serum, microalgae samples, and oxPUFA

The LC-MS/MS data of the microalgae and serum samples was processed as well as the oxPUFA samples with MZmine3 using the same parameters. The features were aligned for each type of sample resulting in two pairs of feature tables and the MS/MS files for the serum and microalgae samples, respectively. Then, a molecular network was constructed employing the GNPS classical molecular network workflow using the extracted MS/MS files from the serum, the microalgae, and the ‘all oxPUFA’ alignment described above. The networking parameters remained consistent with those used for the feature-based molecular network, with the addition of the ‘MS cluster OFF’ (i.e., it is therefore, a feature-based molecular network) option and library matching against both GNPS and our NEO-MSMS libraries.

## Data Records

All the data reported in this article is available in the Zenodo repository under a CC BY 4.0 license as a compressed.zip file with different subfolders^43^. The folder ‘libraries’ contains the NEO-MSMS library divided into three.mgf files, i.e., the standards obtained with Q-Exactive Focus (i.e. NEOMSMS_QFocus_lib.mgf), Orbitrap ID-X Tribrid (i.e. NEOMSMS_idX_lib.mgf), and from the Watrous *et al*. study^7^ (NEOMSMS_Watrous_lib.mgf). Moreover, the individual.mgf files from each compound was included in three subfolders following the same syntax. The folder ‘oxPUFAs’ contains the raw.mzML files of the LC-MS/MS analysis and the MZmine3 (version 3.7.0) alignment results (i.e., the feature tables and the extracted.mgf files). The folder ‘algae_serum’ contains the same type of data from the microalgae and serum samples. The folder ‘oxOxylipins’ contains the raw.mzML files of the LC-MS/MS analysis of the miniaturized oxidation of HETE, rearrangement of PGH_1_ and PGH_2_. Finally, the ‘putative_lib´ folder contains the putatively annotated oxylipins in the same structure as ‘libraries’ folder. All libraries in.mgf format are compatible with GNPS^8^ and MZmine3^40^. Also, the.mzML files of the different experiments as well as the.mgf files of the standards are available in MASSIVE as a GNPS dataset under CC0 1.0 Universal license^44^.

## Technical Validation

The validation of the molecular networking of NEO-PUFAs was accomplished through four distinct strategies. Firstly, the topology of the molecular network generated using the NEO-MSMS standards as input was examined to determine if the uploaded MS/MS data could highlight structural similarities among the standards present in the dataset.

Next, we generated four feature-based molecular networks for each PUFAs, encompassing all types of oxidation. Additionally, a feature-based network was established, including all four fatty acids, to identify potential compounds based on observed connections within the molecular network from which was created our putative library. Moreover, the examination of oxPUFA data played a crucial role in identifying specific putative compounds which were subsequently added to the putative library.

Finally, the dereplication efficiency of the NEO-MSMS oxylipins dataset implemented in GNPS libraries was estimated by annotating the molecular network obtained from blood serum and microalgae extract, and the oxPUFA data large oxylipins/NEO-PUFAs coverage was revealed by highlighting related oxylipins.

### Cartography of classical molecular network of oxylipin standards library

**Fig. 2:**
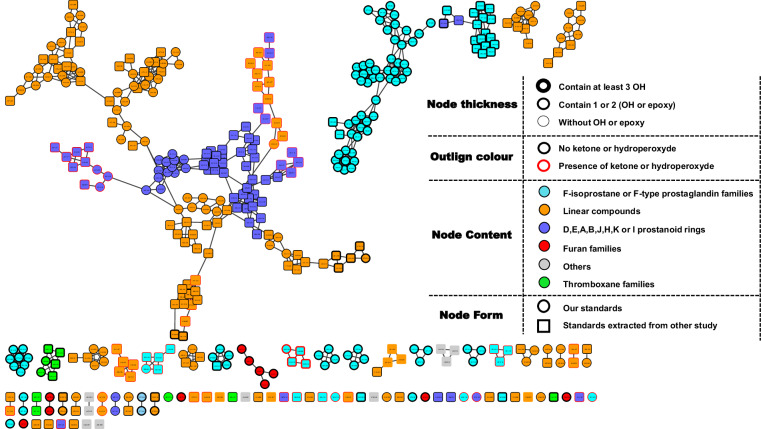
Cartography of the molecular network obtained using the oxylipins/NEO-PUFAs library as an input.

In Fig. 2 is described a classical molecular network that includes all available standards analyzed on the Orbitrap IDx and the Q-Exactive Focus, presented in circular-shaped nodes, along with the standards extracted from the study by Watrous *et al*.^7^, presented in rectangular-shaped nodes. Therefore, this molecular network represents all compounds from the NEO-MSMS library, and their organizational hypotheses which will be used during annotation. Three ways that allow us to classify the compounds can be observed depending on the relationships between structures. Firstly, the compounds are classified by families. For example, it can be observed the F families of isoprostanes^45^ or prostaglandins colored in cyan and grouped together. The linear compounds (non-cyclic) in orange are linked together by series, while the remaining prostaglandin families of types A, B, J, E, D, K, H, or I are classified separately and presented in violet color.

Secondly, on the other hand, the classification of these compounds is also influenced by the number of hydroxyl groups (OH) present in the molecule, excluding the acidic part. For instance, although 13, 14-dihydro-19-hydroxy-PGE_1_ belongs to the E family, it is found within the cluster of F families (top right corner). This can be attributed to the presence of three free OH groups in this molecule leading to fragments resembling the F family.

Lastly, the presence of ketone or hydroperoxide forms, indicated by red color contours (middle left corner in violet). Oxylipins/NEO-PUFAs with 18 Da difference will likely be linked in a molecular network if they are either lipid-hydroperoxide and its corresponding ketone. Similarly, because of similarity in fragmentation patterns, it is also the case for D/E-PG derivatives and their corresponding A/J-PG like isomers.

### Feature-based molecular networks of oxPUFAs

#### NMBHA oxidation

**Fig. 3:**
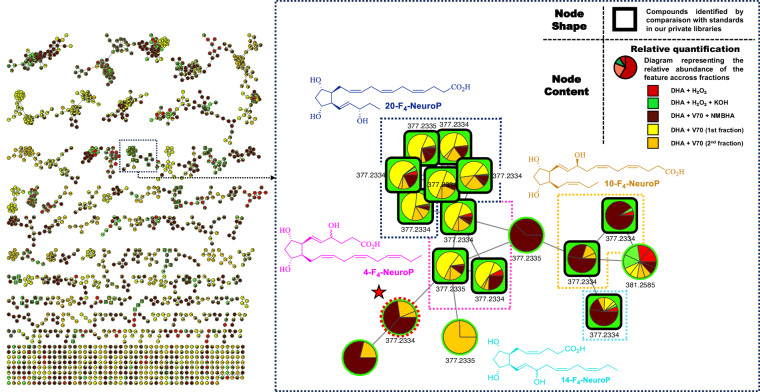
Feature based molecular network of all the DHA oxidation (with zoomed cluster of F_4_-NeuroPs).

A FBMN was created for each fatty acid containing the different studied oxidation. As indicated in the key (Fig. 3), we can examine the distribution of the several DHA oxidation performed: the red colour corresponds to oxidation 1 (H_2_O_2_), green corresponds to oxidation 2 (H_2_O_2_ followed by KOH), yellow and orange represent the first and second fractions of oxidation 3 (V70), while brown represents the only fraction collected with oxidation 4 (V-70 and NMBHA) as the last fraction contaminated with NMBHA was discarded. The squared bold shape are compounds annotated by our NEO-MSMS library.

The FBMN analysis of oxidized DHA, or individually oxidized PUFA, can revealed some rational clustering trends accordingly to families of oxylipins, because whatever the series within a family, a fragmentation pattern remains. For example, the zoomed cluster in Fig. 3, thanks to the library matching using the NEO-MSMS, allowed the annotation of four series of the F_4_-NeuroP family: 20-F_4_-NeuroP, 4-F_4_-NeuroP, 10-F_4_-NeuroP, and 14-F_4_-NeuroP. The several detected stereoisomers per series of NeuroPs are represented by the multiple nodes allowing a rapid dereplication knowing the similarity in fragmentations for diastereomeric compounds, but difference in retention time thanks to FBMN. The interconnection of the nodes can be explained by the fragmentation patterns of the F-series isoprostanoids (or PGs) which is well studied in ESI mode (see Supplementary Information Figure S2)^46^.

Another interesting feature, revealed by the pie charts of such compounds is the NMBHA radical H-atom donor effectiveness at competing with cyclisation processes. The relative quantitative differences in the formation of series of NeuroPs are known to be a direct consequence of further cyclization leading to dioxolane-NeuroPs^47^. In autoxidative condition the 4- and 20-NeuroPs are the major compounds because they cannot further formed 1, 2-dioxolane to the contrary of the other series. However, when NMBHA is used, it reduces the possibility of dioxolane formation, hence the observation of quantitatively higher level of 10 and 14-F_4_-NeuroPs at the detriment of 4- and 20-F_4_-NeuroPs.

Dereplication and finding “novel” features (Fig. 3) is also indicative of the power of molecular networking approach for finding related metabolites. The few non-annotated nodes in this zoomed cluster, like explained above, are potential putative F-type NeuroP. For example, if we take this unknown node with the same precursor mass as F_4t_-NeuroP (highlighted with a star), it is relatively easy to find the missing link as the two main fragments (outside the redundant fragments described in Supplementary Information Figure S2) are m/z 113.0608 and m/z 141.0557 indicative of the 7-series^47^ (Supplementary Information Figure S3). Thus, several compounds have been putatively annotated with a F-prostane ring including DHA, EPA, AA, and ALA precursors.

#### Phosphite reduction

Trimethyl phosphite or triphenyl phosphite is known to reduce hydroperoxide derivatives and also interestingly to selectively reduce the isobaric NEO-PUFAs bicyclic-endoperoxides (like PGG) versus the more stable linear endoperoxides (1, 2-dioxolanes)^48^. It allowed to potentially locate those functions and/or structures (PGG like) in clusters of metabolites (Supplementary Information Figure S4).

#### All oxPUFA

**Fig. 4:**
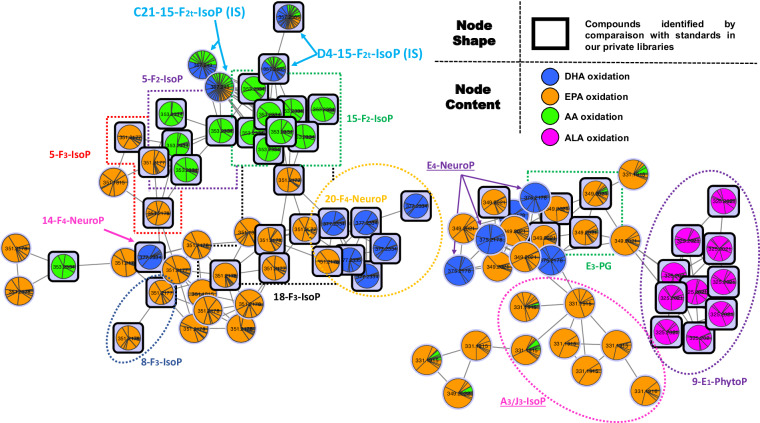
Some clusters from the feature based molecular network from all oxidized PUFA.

As described above, the molecular networking analysis of individual oxidized PUFA is capable of isolating cluster of family of NEO-PUFAs, with the potential of further annotating remaining series. We have established a feature-based molecular network incorporating all the analyzed oxidized PUFAs through the alignment of the four previously used feature tables (mimicking real samples), allowing us to establish new connections between series of different PUFA precursors from the same families. For instance, (Fig. 4, right corner), automatic dereplication using the NEO-MSMS library highlighted the cluster comprising the E- or D-type NEO-EPA (thanks to the identification of PGE_3_, or its likely isoprostanoid diastereoisomer at m/z 349.2021, orange nodes), 9-E_1_-PhytoP derivatives (from ALA of m/z 325.2021, violet nodes), as well as E- or D-type DHA derivatives (m/z 375.2177, blue nodes) and A-or J-type EPA metabolites (m/z 331.1915, orange nodes). It is then possible to annotate some of the detected E_4_-and A_3_-IsoPs based on their MS/MS fragmentations, as 4-E_4_-NeuroP and 17-E_4_-NeuroP (Supplementary Information Figure S5 and S6). Furthermore, 15-A_3_-IsoP was easily identified as directly connected to PGE_3_ (almost identical fragmentation pattern, Supplementary Information Figure S7)^49^.

These inter-family relationships have superseded the intra-family series connections obtained from a single oxidized PUFA. Other clusters have maintained the connection between series of the same family, as is the case in the F_3_-IsoP, F_2_-IsoP and F_4_-NeuroP clusters (Fig. 4).

### Structural determination of oxylipin oxidation products

#### 15-HETE Oxidation

To retrieve data of previously identified NEO-PUFAs (NMR and/or MS/MS) generated by oxidation of peculiar oxylipins, we miniaturized the original V-70 radical initiated oxidation protocol developed by Rector *et al*.^32^. Applied on 15 HETE, it permitted to detect and acquire corresponding diol metabolites, of which 4 were elucidated as: the well-known 5, 15-DiHETE and 8, 15-DiHETE (as their commercially available enzymatic isomers), and the rediscovered 9, 15-DiHETE and 14, 15-DiHETE (Supplementary Information Figures S8–S11). Those NEO-PUFAs represented 4 out of the 7 leukotriene-like metabolites reported (the non-use of NMBHA and the non-reduction procedure could explain the lower number of diols recovered).

#### PGHs transformation into levuglandins

**Fig. 5:**
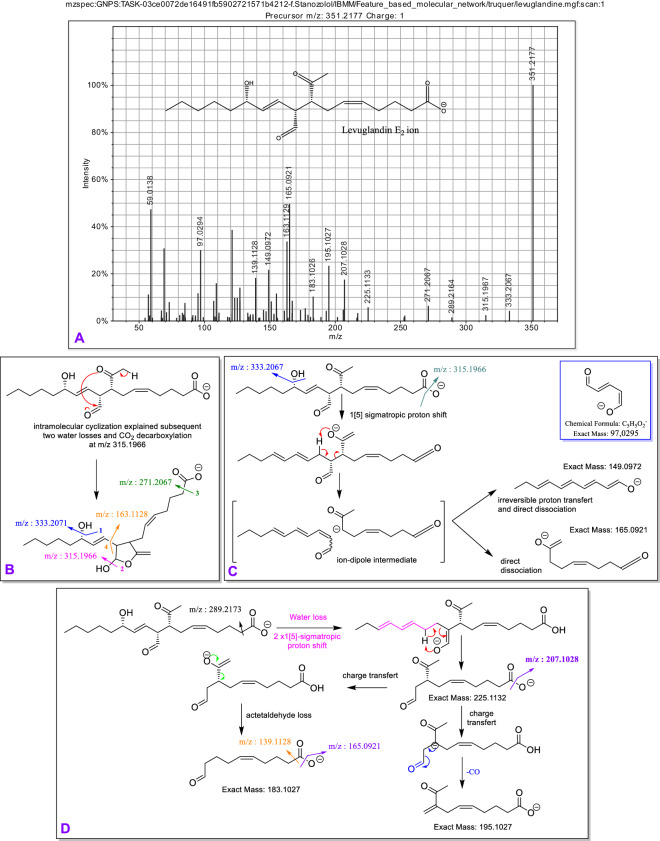
Electrospray ionization (negative ions) and tandem mass spectrometry of putative Levuglandin-LGE_2_ obtained from the rearrangement of PGH_2_ and some putative characteristic fragmentation mechanisms. (**A**) MS/MS spectrum used for the identification (**B**) Intramolecular cyclisation of the ketoaldehyde unit produces a lactol derivative explaining the otherwise inextricable double water losses and decarboxylation. (**C**) Ion-dipole intermediate generated after double water losses can explain characteristic fragments m/z 149.0972, 165.0921. (**D**) Water loss and double 1[5]-sigmatropic proton shift followed by H-ene reaction led to characteristic fragments m/z 183.1027 after acetaldehyde loss, and m/z 195.1027 after CO loss.

The elusive MS/MS data of levuglandins and other iso-levuglandins prompted us to perform the protocol described by Salomon *et al*. that could smoothly transform PGHs into levuglandins (LGE) via the Kornblum-DeLaMare rearrangement^29^. Accordingly, as described in that seminal paper, several compounds were identified like the corresponding PGF, PGD, PGE, keto-PG (validated by rt and MS/MS from standards, Supplementary Information Figures S12–S16), and ultimately what we putatively assigned as levuglandin-LGE. The given away fragments for LGE_2_ are the ones in between the two carbonyl functions (m/z 183.1027, m/z 149.0972, m/z 225.1132, m/z 195.1027 and 165.0921 on Fig. 5). Once more, microscalling PUFAs oxidation or transformation is feasible and allows for the retrieval of MS/MS data of previously discovered NEO-PUFAs missing such valuable information.

### Oxylipins/NEO-PUFAs dereplication in blood serum and microalgae samples with oxPUFA and NEO-MSMS

To assess the NEO-MSMS library’s annotation capability with real samples, we initially utilized the ‘Spectral Library Search’ workflow within GNPS^9^. We used the.mgf files obtained following the preprocessing of serum and algae samples using MZmine3 described in the Methods section. The matching parameters were 0.6 minimum cosine score, at least 5 matching peaks, 0.02 Da precursor error, and 0.02 Da product ion error. Given these parameters, the results will be as follows:

During the serum analysis, a total of 190 compounds were successfully annotated using the NEO-MS/MS library, as indicated in Supplementary Table S4. Similarly, in the microalgae extract analysis, 138 compounds were annotated, as documented in Supplementary Table S5. These annotations account for 45.2% and 50.5% of the overall annotated compounds for serum and microalgae, respectively.

**Fig. 6:**
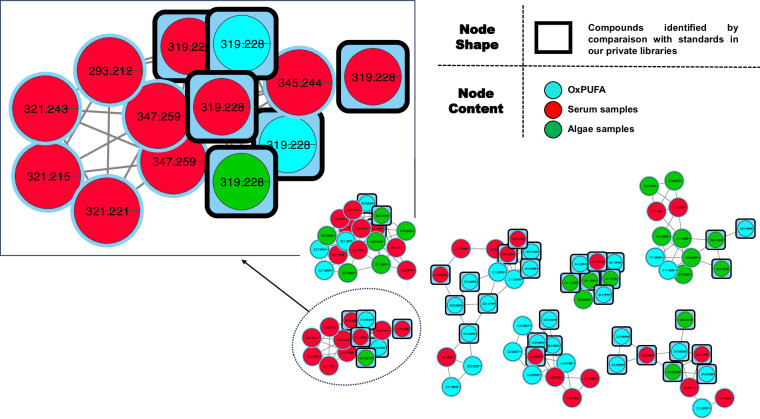
Example of some clusters from the molecular network from all oxidized PUFA with serum and algae samples.

Following the validation of our library, we established a classical molecular network that incorporated MS/MS data from the aligned oxPUFAs features (see all oxPUFA), serum samples, and algae samples, as illustrated in (Fig. 6). For the sake of reusability of oxPUFA data for the community, we chose classical molecular network workflow since FBMN would come short of utility in this case as the alignment procedure is machine/sample dependent. Our oxPUFA data, depicted by the blue nodes, exhibit strong connections to nodes exclusively found in the serum extracts (depicted in red) and algae (depicted in green), indicating that oxPUFA data is particularly effective in discerning other NEO-PUFAs as the ones already present in oxPUFA data (potentially originating from other fatty acids).

An example from the zoomed-in cluster (Fig. 6) showed different nodes annotated as 11-HETE employing NEO-MSMS library (i.e., the black squares of m/z 319.228 in the Fig. 6) which potentially correspond to different isomers due to the difference on the retention times (max. difference of 0.09 min). These nodes are linked with another two with virtually identical mass spectra (cosine score 0.9929), m/z 347.259 (C_22_H_35_O_3_), which could correspond precisely to a monohydroxyl derivative of a fatty acid with the formula C_22_H_36_O_2_. Similarly, the node with m/z 345.244 (C_22_H_34_O_3_) may correspond to a monohydroxyl derivative of C_22_H_34_O_2_, and m/z 293.2122 (C_18_H_29_O_3_) to the monohydroxyl derivative of C_18_H_28_O_2_.

The position of the hydroxyl group relative to the end of the chain plays a crucial role in connecting compounds of the same family. For example, an OH group at position 11 of a C20 can easily be linked to a monohydroxyl compound with an OH group at position 13 of a C22, or an OH group at position 9 of a C18. The study of the MS/MS spectrum clearly demonstrated that the compound with m/z 347.259 corresponds to 13-hydroxy-7Z, 10Z, 14E, 16Z-docosatrienoic acid (13-HDT) a metabolite of adrenic acid (the 16^th^ isomer position could potentially be E). This is supported by the specific fragments at m/z 195 and m/z 223, as shown in Supplementary Information Figure S17.

Regarding the compound with m/z 293.2122 (C_18_H_29_O_3_), the 9-monohydroxyl of linolenic acid (C_18_H_28_O_2_) was initially investigated but two additional Da on the specific major fragments led us to consider other isomers of ALA, such as pinolenic acid. The putative fragments of 9-hydroxy-(5Z, 10Z, 12Z)-octadeca-5, 9, 12-trienoic acid would match with the observed spectrum (Supplementary Information Figure S18). The third node with m/z 345.244 must correspond to the 13-monohydroxyl of Osbond acid (13-hydroxy (4Z, 7Z, 10Z, 14E, 16Z)-docosapentaenoic acid), rather than clupanodonic acid, in order for the major fragments to be explained properly (Supplementary Information Figure S19). The two compounds, 13-HDT and the 9-monohydroxyl of pinolenic acid, are in turn connected to one node with masses m/z 321.243 (C_20_H_33_O_3_), likely a monohydroxyl of dihomo-gamma-linolenic acid C_20_H_34_O_2_ (Supplementary Information Figure S20). The plausible characteristic fragments of the 11-hydroxy-(8Z, 12E, 14Z)-icosa-8, 11, 14-trienoic acid correspond perfectly to those observed in the spectrum.

These ‘novel oxylipins/NEO-PUFAs’ arising from enzymatic or non-enzymatic processes would be completely overlooked if only a targeted analysis is to be performed, questioning the utility of adapting a targeted MRM solution based on oxPUFA data^50^. The use of oxPUFA data along with our NEO-MSMS library can be highly valuable, both for annotating compounds found in biological matrices and for connecting new structures to discover potential novel biomarkers. The very precise nature of OxPUFA data also likely suggest its use for machine-learning based annotation^51^.

### Dereplication of the NEO-MSMS against the “putative Watrous oxylipins” available on the MASSIVE repository

In the Watrous studies^7,52^, more than 500 “unknown or putative oxylipins” were discovered, and as initially hypothesized in this manuscript and one of the purpose for this NEO-MSMS library, we believed that some of them could be related to NEO-PUFAs. We subsequently reanalyzed the data presented by Watrous *et al*. in 2019 through a conventional molecular network, using the same parameters and incorporating our library NEO-MSMS. However, the MS/MS files available present a mass error that can exceed 0.04 m/z and could not be used to match lipid datasets for predicting known fragmentations (simple loss of water or CO_2_ are already off target to match any formulae of oxylipins). Nevertheless, the power of molecular networking is that by increasing the mass error tolerance to 0.1 instead of 0.02, we could carry out dereplication. However, still due to the complexity of further analysis of the data, we specially focused on three specific oxylipins; i.e, oxylipin 1, 2, and 3 (named as such in the original text) which showed significant changes after one-year PUFA n-3 treatment (VITAL study) and associated with clinical lipid and inflammatory biomarkers^52^. We also looked at five other unidentified putative compounds from the original Watrous *et al*. study which were frequently found to be associated with multiple correlates of inflammation, while other putative compounds, were specifically linked to age, BMI, or CRP levels, suggesting both universal and specific inflammation mediators^7^.

As described (in the Supplementary Information Figs. S21), 6 out of the 8 putatively assigned oxylipins (vide supra) could be annotated with great confidence as NEO-PUFA (isomer of 5-HETE, 9-*epi*-9-F_1t_-PhytoP, isomer of 20-HDHA, 11-hydroxy-7E, 9Z, 13Z, 16Z-docosatrienoic acid (11-HDT), 13-hydroxy-7E, 10E, 14E, 16Z-docosatrienoic acid (13-HDT) and 14, 17-DiHEPE) and not as enzymatic oxylipins, thanks to the NEO-MSMS associated with the library building of GNPS and the molecular networking facility of GNPS using oxPUFA data.

We validate that NEO-MSMS and oxPUFA data could help reinterpreting the previously identified families of NEO-PUFAs of which data were not collected at the time. It could also help the isolation of formerly unreported metabolites.

## Usage Notes

The NEO-MSMS library can be used directly in the GNPS environment for the different molecular networking analysis. Also, it is available as a set of.mgf files compatible with MZmine3 libraries. oxPUFA files could be used for conducting classical molecular networking analysis with another samples as we demonstrated for serum and algae samples and dereplicate oxylipins. Additionally, the oxPUFA files will also be located in the MASSIVE repository as GNPS dataset allowing their use with GNPS MASST^53^. With this tool, the oxPUFA data can be re-analyzed and used for the annotation of unknowns as putative NEO-PUFA in future lipidomic studies with special value for the novel separation approaches employing sophisticated chiral LC^54^ or ion mobility^55,56^.

### Supplementary information

Supplementary Information

Supplementary Tables

## Data Availability

No custom code has been used in the generation of this work.
