# Supplementary material for: From MS/MS library implementation to molecular networks: Exploring oxylipin diversity with NEO-MSMS
Source: Sci Data. 2024 Feb 13;11:193. doi: 10.1038/s41597-024-03034-4 (PMC10864323; doi:10.1038/s41597-024-03034-4)
Supplement: Supplementary file 1 — Supplementary Information [file 41597_2024_3034_MOESM1_ESM.pdf]

Supplementary Information

From MS/MS Library Implementation to Molecular Networks: Exploring Oxylipin Diversity with NEO-MSMS

Authors

Anis Elloumi[1], Lindsay Mas-Normand[1], Jamie Bride[2], Guillaume Reversat[1], Valérie Bultel-Poncé[1], Alexandre Guy[1], Camille Oger[1], Marie Demion[2], Jean-Yves Le Guennec[2], Thierry Durand[1], Claire Vigor[1], Ángel Sánchez-Illana[1,3]*, Jean-Marie Galano[1]*

Affiliations

1. Institut des Biomolécules Max Mousseron (IBMM), UMR 5247-CNRS, 34293 Montpellier, France
2. PhyMedExp, Université de Montpellier, Inserm U1046, UMR CNRS 9412, Montpellier, France
3. Department of Analytical Chemistry, University of Valencia, Dr. Moliner 50, 46100 Burjassot, Spain

* corresponding author(s): Jean-Marie Galano (jean-marie.galano@umontpellier.fr) and Ángel Sánchez-Illana (angel.illana@uv.es)

Table of Contents

Supplementary Figure S1. ^{1}H NMR investigation of crude reaction mixture, and partial purification. 3

Supplementary Figure S2. Detailed explanations of the redundant fragments for F-type isoprostanoid/prostaglandin based on fragmentation patterns of the 20-F_{4t}-Neuroprostane under electrospray ionization (negative ions). 4

Supplementary Figure S3. Electrospray ionization (negative ions) and tandem mass spectrometry of putative 7-F_4-NeuroP and a mirror view of the 10-F_{4t}-NeuroP vs 7-F_{4t}-NeuroP. 5

Supplementary Figure S4. Some cluster from the feature based molecular network from oxidized DHA with and without reduction by trimethyl phosphite. 7

Supplementary Figure S5. Electrospray ionization (negative ions) and tandem mass spectrometry of 17-E_4-NeuroP obtained from the oxidation of the DHA. 8

Supplementary Figure S6. Electrospray ionization (negative ions) and tandem mass spectrometry of 4-E_4-NeuroP obtained from the oxidation of DHA. 9

Supplementary Figure S7. A mirror view of the PGE_3 standard electrospray ionization (negative ions) and tandem mass spectrometry vs the putative 15-A/J_3-IsoP obtained from the oxidation of EPA. 10

Supplementary Figure S8. Electrospray ionization (negative ions) and tandem mass spectrometry of 5,15-DiHETE obtained from the oxidation of the 15-HETE. 11

Supplementary Figure S9. Electrospray ionization (negative ions) and tandem mass spectrometry of putative 9,15-DiHETE obtained from the oxidation of the 15-HETE and fragmentation mechanisms. 12

Supplementary Figure S10. Electrospray ionization (negative ions) and tandem mass spectrometry of putative 8,15-DiHETE obtained from the oxidation of the 15-HETE. 14

Supplementary Figure S11. Electrospray ionization (negative ions) and tandem mass spectrometry of 14,15-DiHETE obtained from the oxidation of the 15-HETE. 15

Supplementary Figure S12. Electrospray ionization (negative ions) and tandem mass spectrometry of PGH_2. 16

Supplementary Figure S13. Electrospray ionization (negative ions) and tandem mass spectrometry of PGE_2 obtained from the rearrangement of the PGH_2. 17

Supplementary Figure S14. Electrospray ionization (negative ions) and tandem mass spectrometry of PGD_2 obtained from the rearrangement of the PGH_2. 18

Supplementary Figure S15. Electrospray ionization (negative ions) and tandem mass spectrometry of PGF_2 obtained from the rearrangement of the PGH_2. 19

Supplementary Figure S16. Electrospray ionization (negative ions) and tandem mass spectrometry of keto-PGE_2 obtained from the rearrangement of the PGH_2. 20

Supplementary S17. Electrospray ionization (negative ions) and tandem mass spectrometry of the compound with m/z 347.259 and the plausible specific fragments of the 13-hydroxy-7Z,10Z,14E,16Z-docosatrienoic acid (13-HDT). 21

Supplementary S18. Electrospray ionization (negative ions) and tandem mass spectrometry of the compound with m/z 293.212 and the plausible specific fragments of 9-monohydroxyl of pinolenic acid : 9-hydroxy-(5Z,10Z,12Z)-octadeca-5,9,12-trienoic acid and plausible specific fragments of the 9-monohydroxyl of alpha linolenic acid : 9-hydroxy-(10E,12Z,15Z)-octadeca-9,12,15-trienoic acid. 22

Supplementary S19. Electrospray ionization (negative ions) and tandem mass spectrometry of the compound with m/z 345.244 and the plausible specific fragments of 13-monohydroxyl of Osbond acid : 13-hydroxy (4Z,7Z,10Z,14E,16Z)-docosapentaenoic acid. 23

Supplementary S20. Electrospray ionization (negative ions) and tandem mass spectrometry of the compound with m/z 321.243 and the plausible specific fragments of 11-monohydroxyl series of dihomo-gamma-linolenic : 11-hydroxy-(8Z,12E,14Z)-icosa-8,11,14-trienoic acid. 24

Supplementary S21. Dereplication of the NEO-MSMS against the "putative watrous' oxylipins" previously available on the GNPS library. 25

References 32

Supplementary Figure S1. ^{1}H NMR investigation of crude reaction mixture, and partial purification.

We have performed ^{1}H NMR attribution of 1,2-dioxolane **1**[1,2] provided from the Ferrié laboratory using the P(OCH$_3$) protocol showing no sign of reduction. In short, approximatively 5 mg of 1,2-dioxolane **1** were dissolved in 5 mL of hexane/EtOH/acetic acid (70:29.4:0.6) and 250 µl of P(OMe)$_3$ was added at room temperature for 1h. Evaporation to dryness was followed by ^{1}H NMR in CDCl$_3$ (400MHz) and showed no sign of removal of the characteristic endoperoxide H-atoms (in green the crude reaction mixture of the reduction protocol, in red the starting material).

Similarly, 5 mg of **1** was dissolved in 5 mL of 1M KOH in water and stirred for 30 min at 40 °C. After cooling, and acidifying the reaction mixture to pH 1 with sulfuric acid (approx.; 1.7 mL), the reaction mixture was extracted with EtOAc twice (10 mL), the combined organic layers washed with brine (5 mL), dried over MgSO$_4$, filtered and evaporated to dryness. ^{1}H NMR in CDCl$_3$ (400MHz) showed no sign of removal of the characteristic endoperoxide H atoms at 4.5-4.2 ppm (in blue the crude reaction mixture of the hydrolysis protocol, in red the starting material).

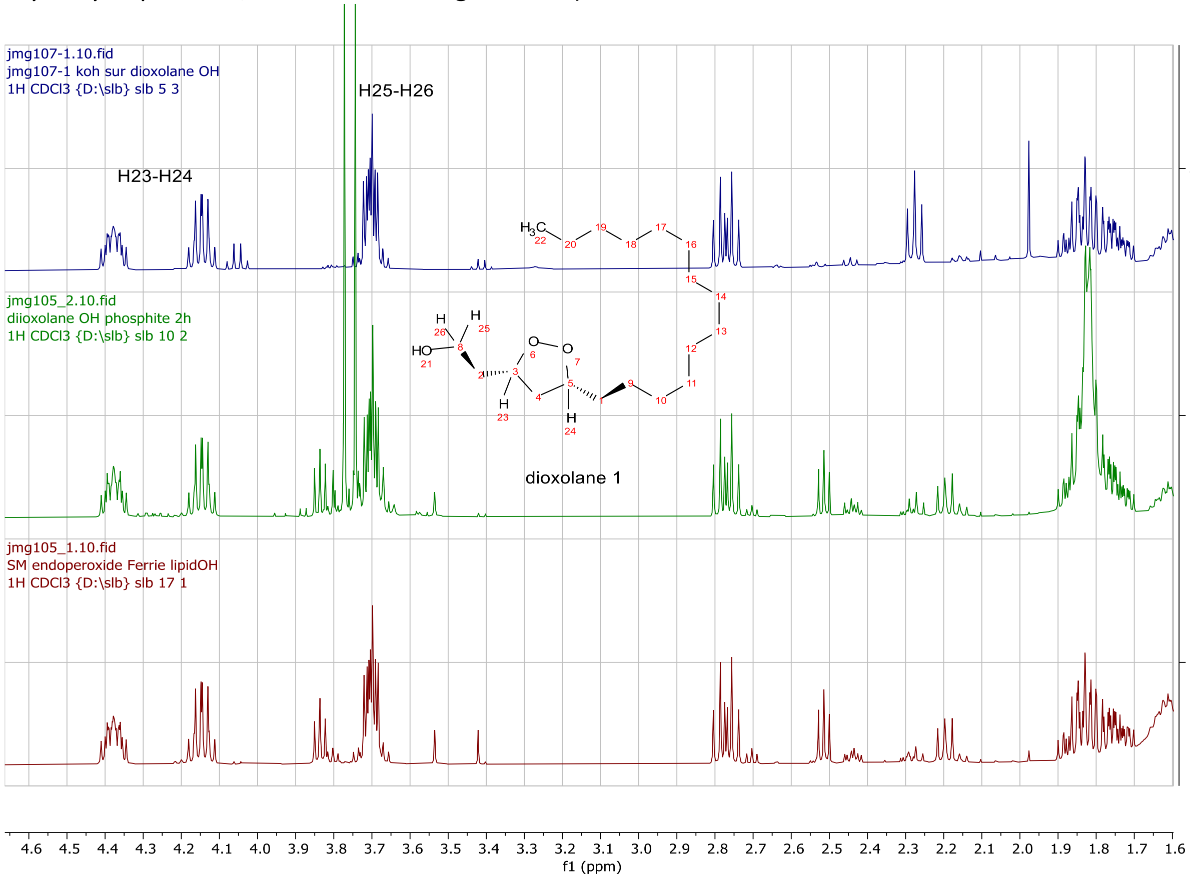

Supplementary Figure S2. Detailed explanations of the redundant fragments for F-type isoprostanoid/prostaglandin based on fragmentation patterns of the 20-F$_{4t}$-Neuroprostane under electrospray ionization (negative ions).

HRMS reveals the loss of acetaldehyde (C_2H_4O) probably resulting from fragmentation of ***deprotomer 1*** precursor ion. This renders F-type (1,3-bishydroxy-cyclopentane unit) isoprostanoid/prostaglandin skeleton easily identifiable to other oxylipins/NEO-PUFAs as this skeleton would never produce a direct loss of CO_2 from precursor ion.

For ***deprotomer** 1* of precursor ion of 20-F$_{4t}$-Neuroprostane after initial acetaldehyde loss is also typically followed by losses of H_2O loss and CO_2. We also noticed (not reported as much as we could look into literature) a CO loss for some particular F-IsoP/PG series (but not all of them).

Deprotomer 2 of precursor ion of 20-F$_{4t}$-Neuroprostane (carboxylate) fragments to loss of H_2O then CO_2 then C_2H_4 (not always the case for other series). After the initial water loss, two consecutive further dehydratation and decarboxylation can occur. However, the second dehydratation will also lead to decarboxylation and water loss.

Regardless of the series being studied, a large majority of the above fragmentations will occur which will represent the some of the spectrometric signature of F-type isoprostanoid/prostaglandin. This explains the recurrence of these molecules within the same cluster.

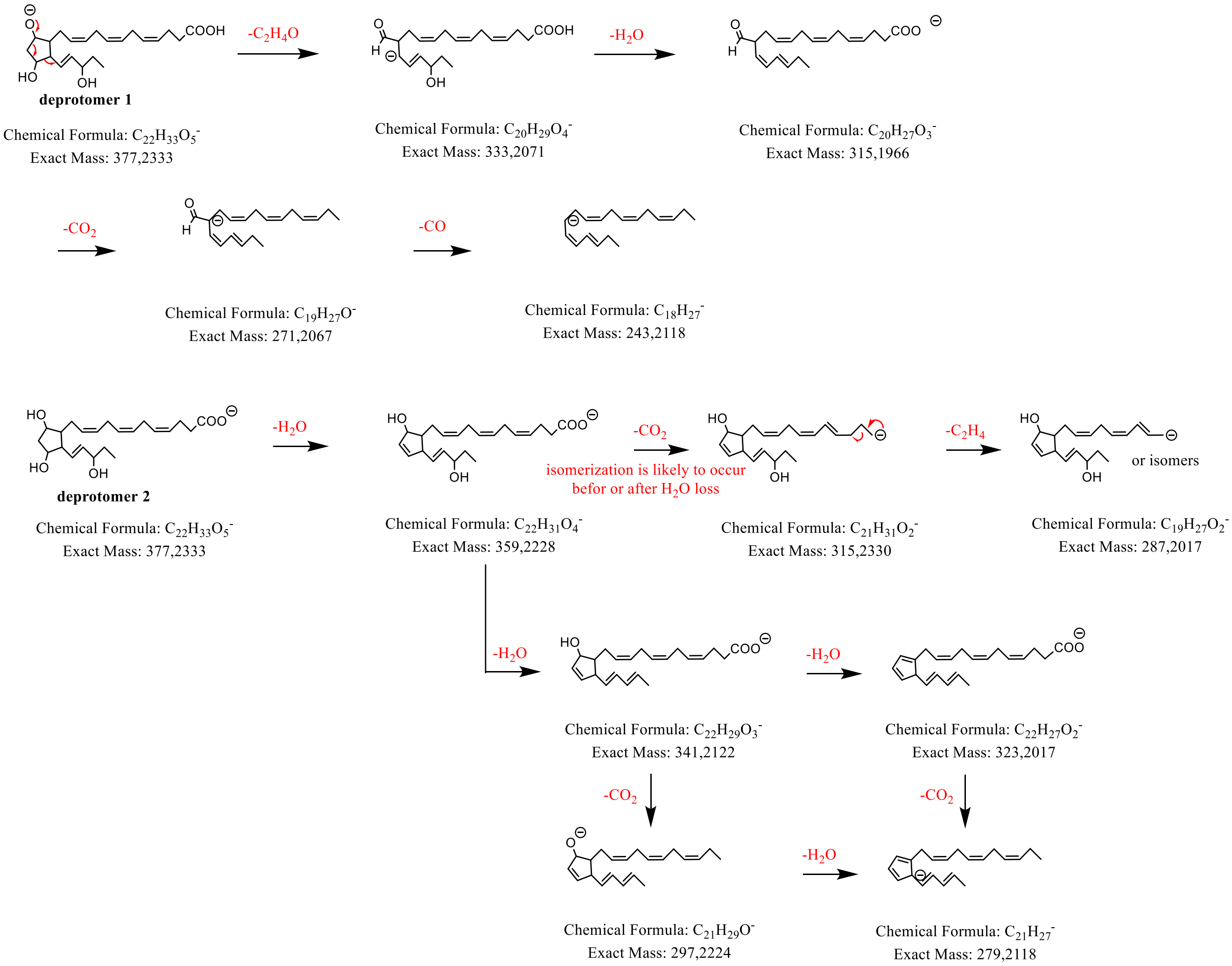

Supplementary Figure S3. **Electrospray ionization (negative ions) and tandem mass spectrometry of putative 7-F_4-NeuroP and a mirror view of the 10-F_{4t}-NeuroP vs 7-F_{4t}-NeuroP.**

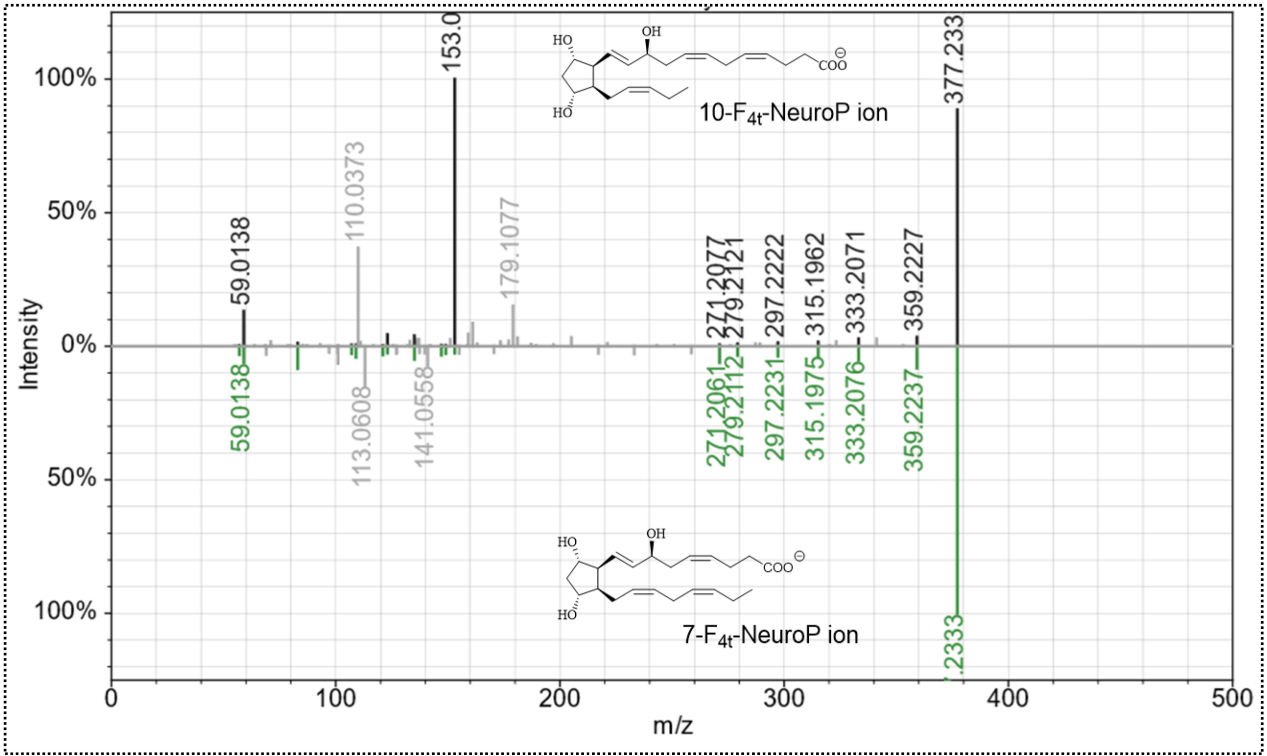

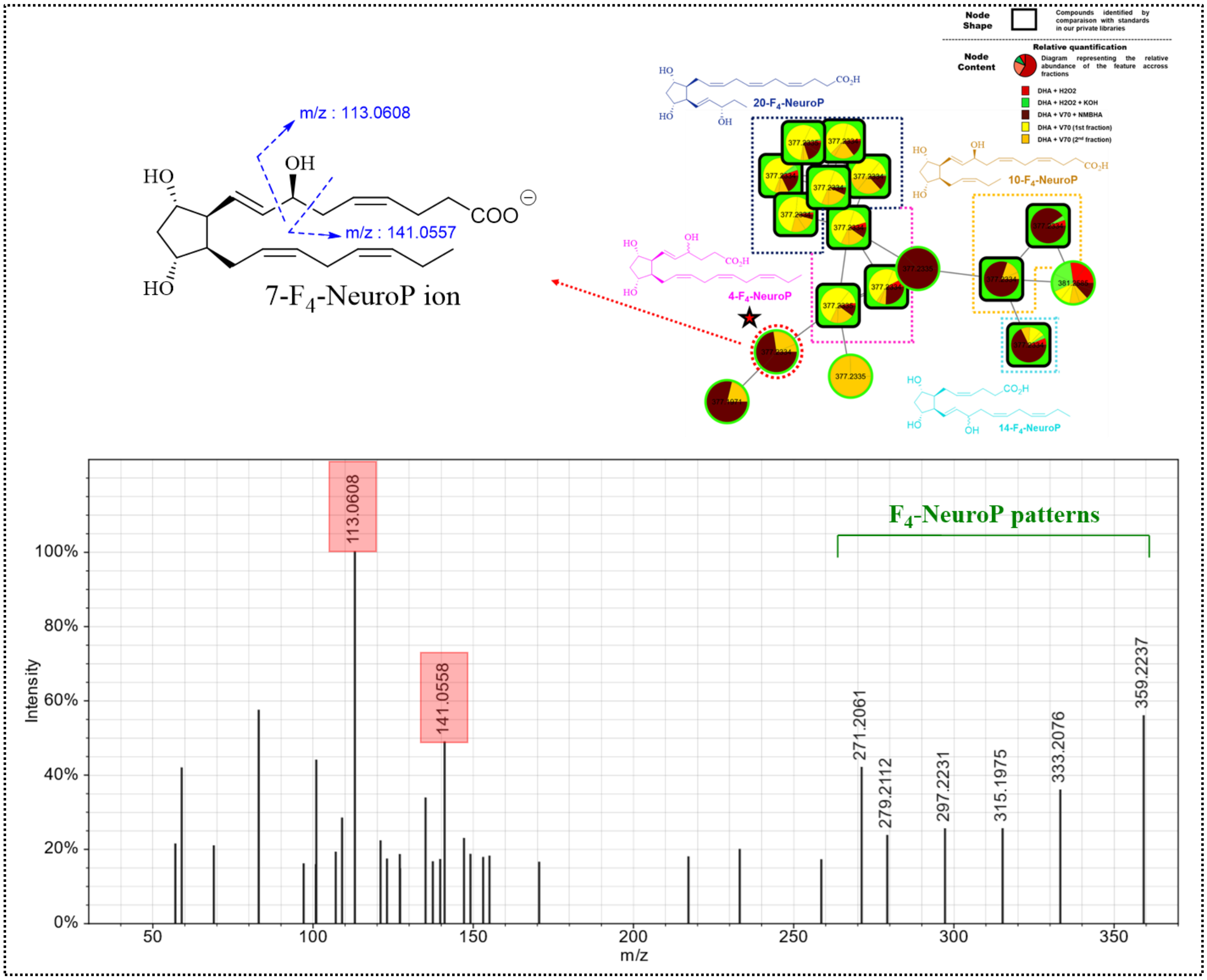

m/z : 113.0608
OH
HO
COO
m/z : 141.0557
HO
7-F4-NeuroP ion
Node Shape
Compounds identified by comparaison with standards in our private libraries
Node Content
Relative quantification
Diagram representing the relative abundance of the feature accross fractions
DHA + H2O2
DHA + H2O2 + KOH
DHA + V70 + NMBHA
DHA + V70 (1st fraction)
DHA + V70 (2nd fraction)
20-F4-NeuroP
10-F4-NeuroP
4-F4-NeuroP
14-F4-NeuroP
377.2335
F4-NeuroP patterns
113.0608
141.0558
271.2061
279.2112
297.2231
315.1975
333.2076
359.2237
Intensity
m/z

Supplementary Figure S4. **Some cluster from the feature based molecular network from oxidized DHA with and without reduction by trimethyl phosphite.**

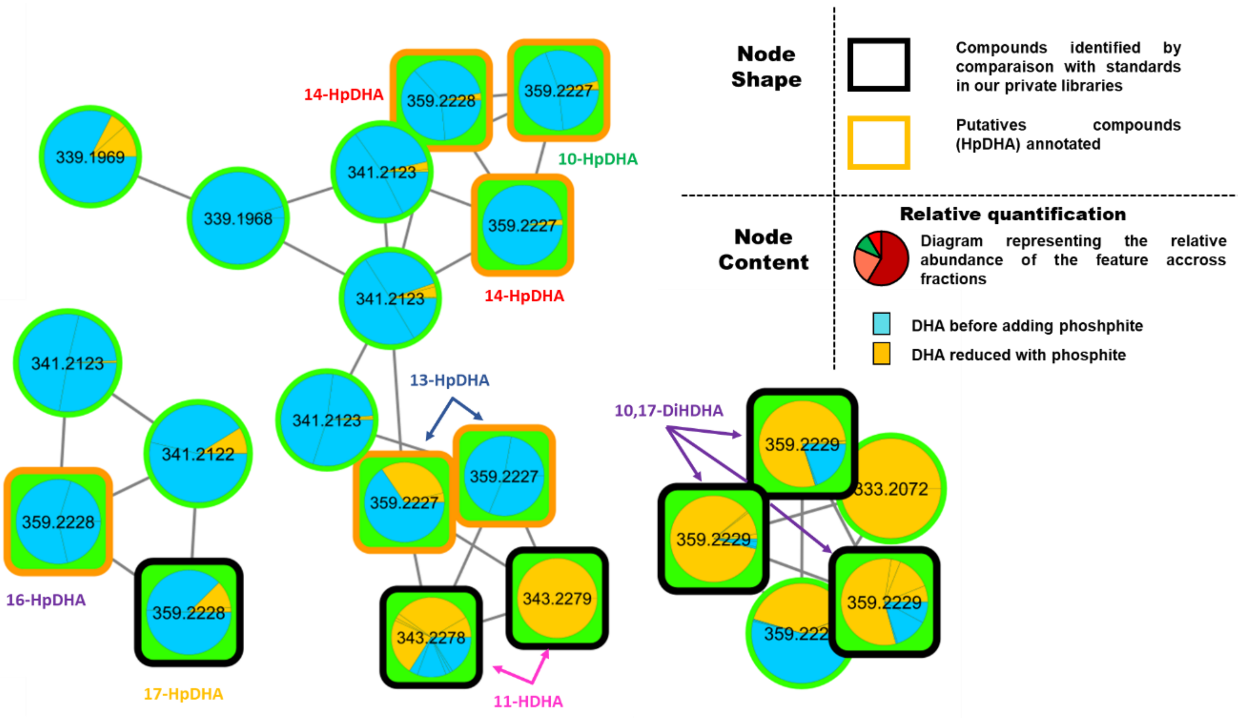

Supplementary Figure S5. **Electrospray ionization (negative ions) and tandem mass spectrometry of 17-E$_4$-NeuroP obtained from the oxidation of the DHA.**

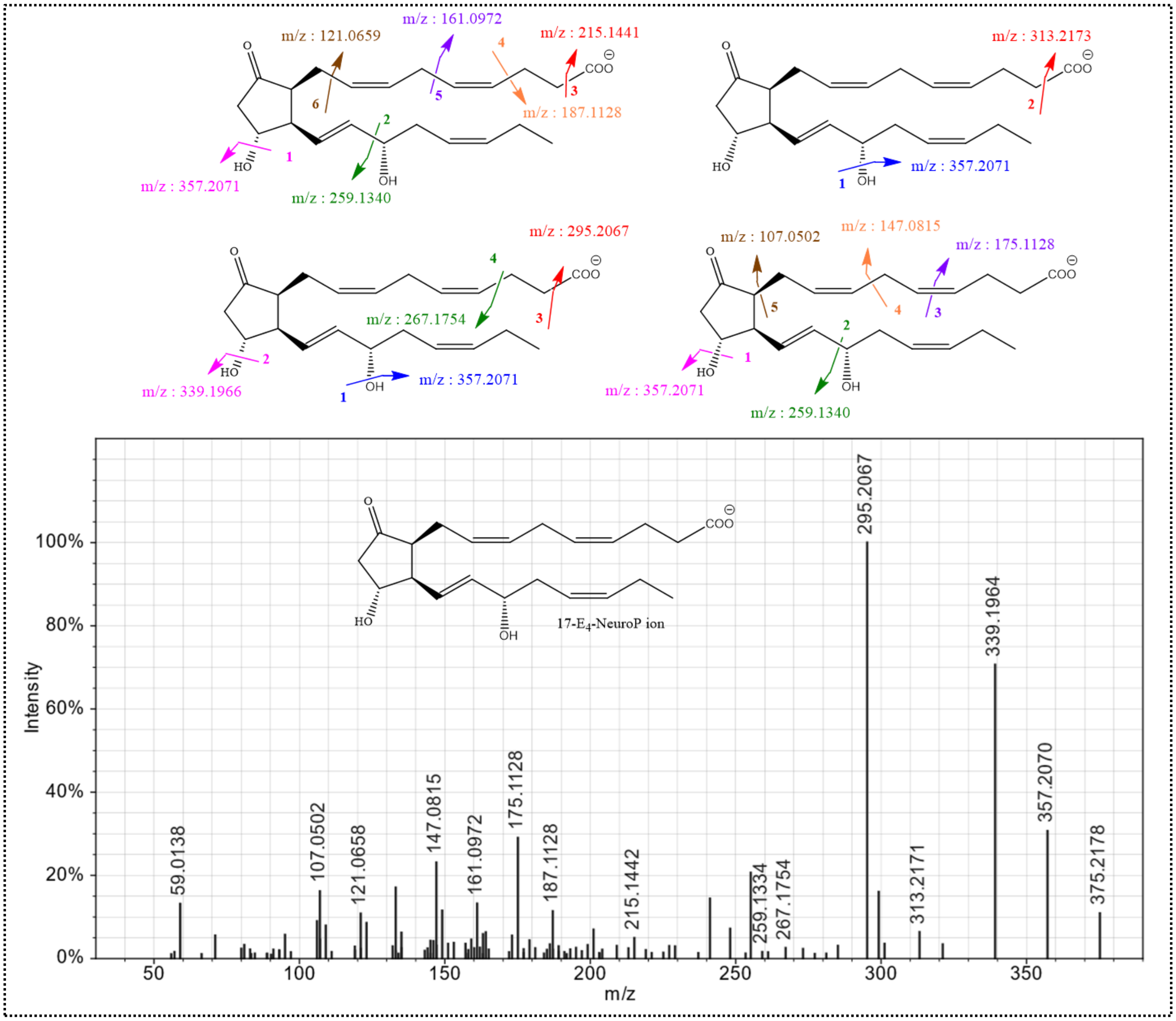

Supplementary Figure S6. Electrospray ionization (negative ions) and tandem mass spectrometry of 4-E_4-NeuroP obtained from the oxidation of DHA.

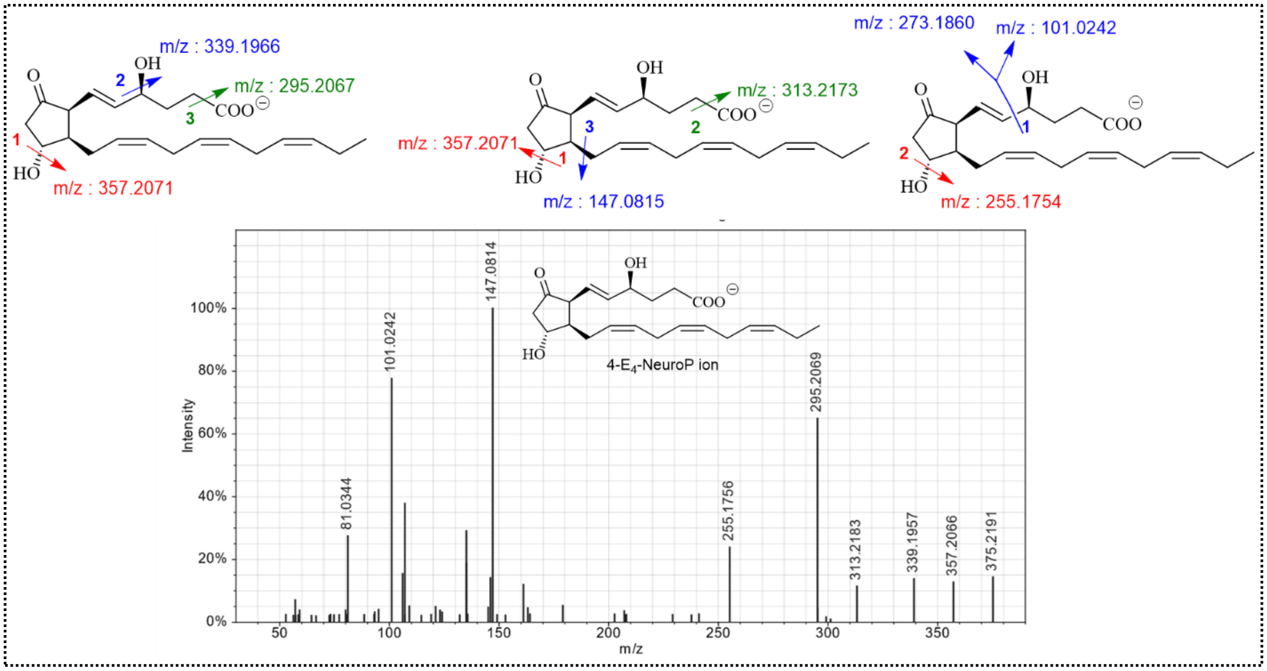

Supplementary Figure S7. **A mirror view of the PGE_3 standard electrospray ionization (negative ions) and tandem mass spectrometry vs the putative 15-A/J_3-IsoP obtained from the oxidation of EPA.**

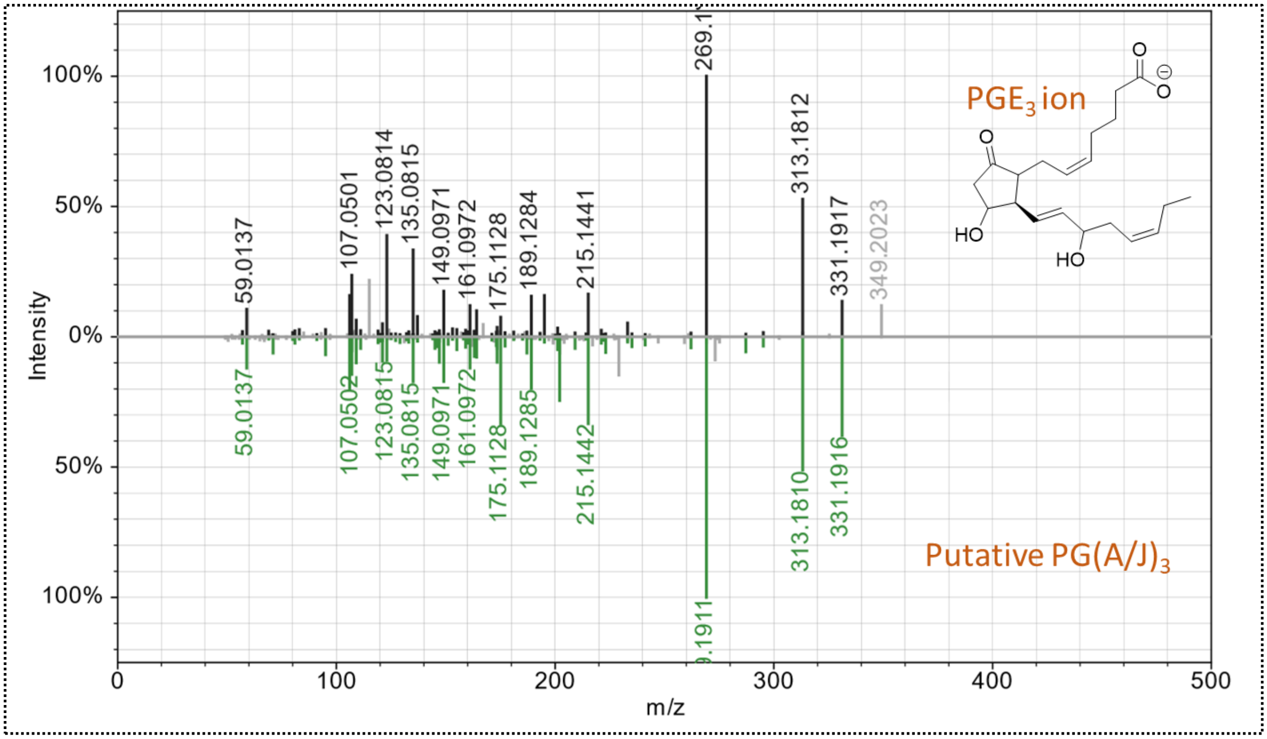

Supplementary Figure S8. Electrospray ionization (negative ions) and tandem mass spectrometry of 5,15-DiHETE obtained from the oxidation of the 15-HETE.

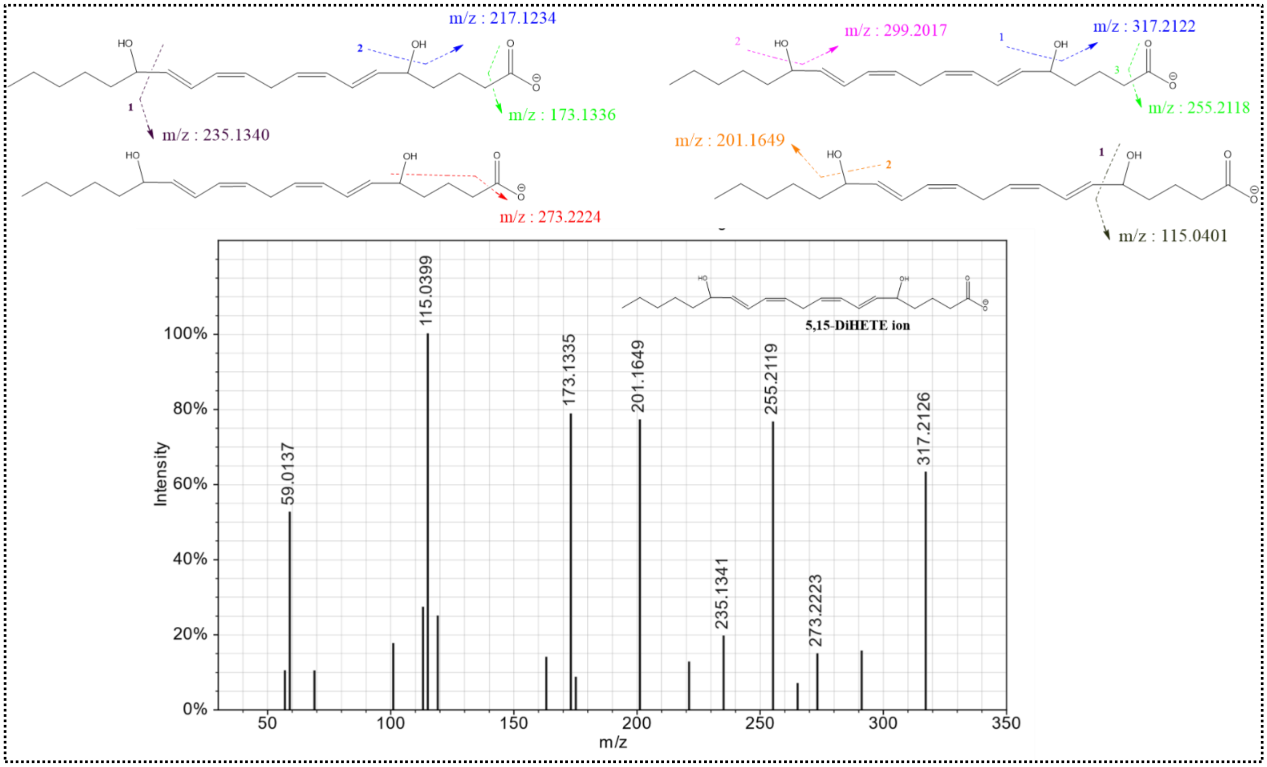

Of note, in the Punta et al.[5] experiment the use of NMBHA avoid the isomerization of the double bonds adjacent where the H-atom is abstracted which occurred because of O_2 peroxyl radical β-fragmentation rate constant competition if no fast H-atom donor is used. Hence, we cannot attribute the stereochemistry of the DiHETEs from Figure 8-11, and they are drawn as the one described in the original paper. It is common sense to remember that we cannot attribute this stereochemistry by MSMS.

Supplementary Figure S9. Electrospray ionization (negative ions) and tandem mass spectrometry of putative 9,15-DiHETE obtained from the oxidation of the 15-HETE and fragmentation mechanisms.

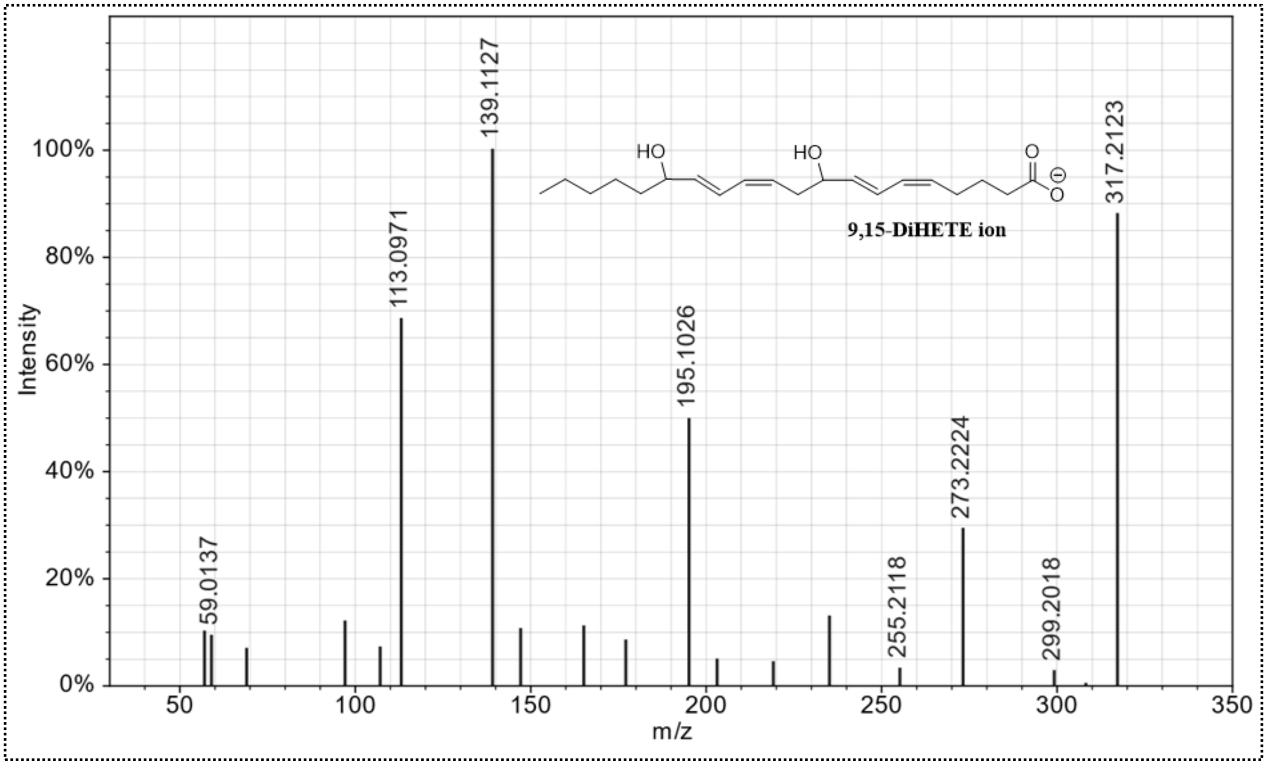

The intriguing fragmentation patterns positioned at the C11-C12 double bond which can be counterintuitive but can be explained by the following fragmentation mechanisms. Other examples have been described in the past for other oxylipins, see for example[3].

For the formation of ion m/z 195.1027, our proposed mechanism involves an initial 1[5] sigmatropic proton shift from C15 to C11, succeeded by charge-driven cleavage of the C11,12 allylic bond. This cleavage results in the simultaneous loss of acetylene (C12-C13), generating an ion dipole intermediate. This intermediate can lead to the direct dissociation forming ion m/z 195.1027. Alternatively, after proton transfer and subsequent direct dissociation, ion m/z 113.0972 is formed. The occurrence of ion m/z 139.1128 can also be explained by the cleavage of the C11,12 allylic bond, resulting in another ion dipole intermediate that produces ions m/z 195.1027 or m/z 139.1128.

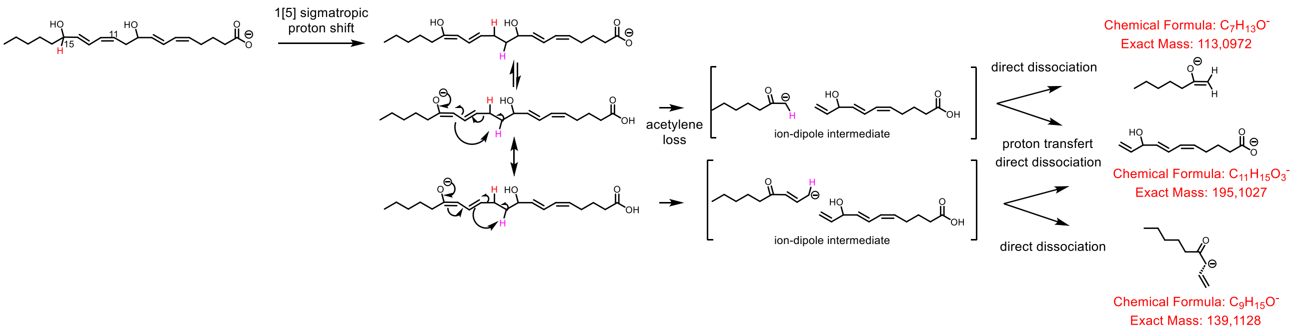

Another fragmentation pathway, based on previously reported rearrangements, is depicted below and it begins with an initial dehydration process, followed by a β-ene rearrangement leading to a typical 1,2-disubstituted-cyclohexadiene intermediate. Subsequently, decarboxylation occurs along with the loss of an ethylene molecule. The resulting ion m/z 245.1911 can rearrange to cleave the CC bond of the cyclohexadiene, followed by proton exchange, resulting in fragmentation and yielding 5 molecules of acetylene, forming ion m/z 113.0972. This overall process is therefore entropically favoured, involving the loss of small molecule gases. Interestingly, all potential β-ene rearrangement ions have the capability to produce m/z 113.0972.

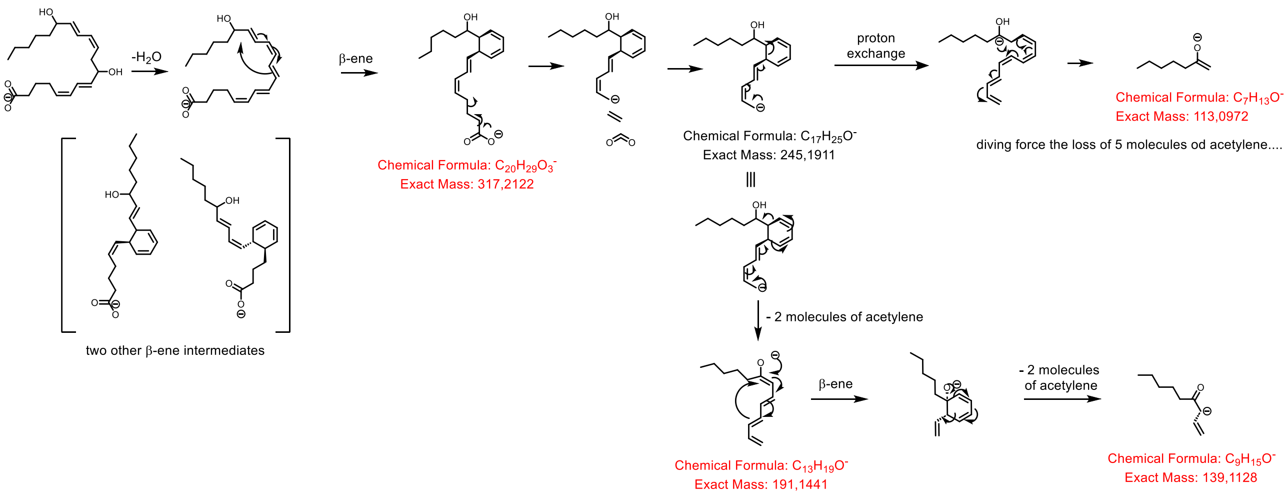

Ion m/z 245.1911 can also rearrange by losing two acetylene molecules, forming the detected ion m/z 191.1441. Subsequent β-ene rearrangement will ultimately produce ion m/z 139.1128 with the loss of two additional acetylene molecules. Once again, the formation of small molecule gases drives this rearrangement process.

Supplementary Figure S10. **Electrospray ionization (negative ions) and tandem mass spectrometry of putative 8,15-DiHETE obtained from the oxidation of the 15-HETE.**

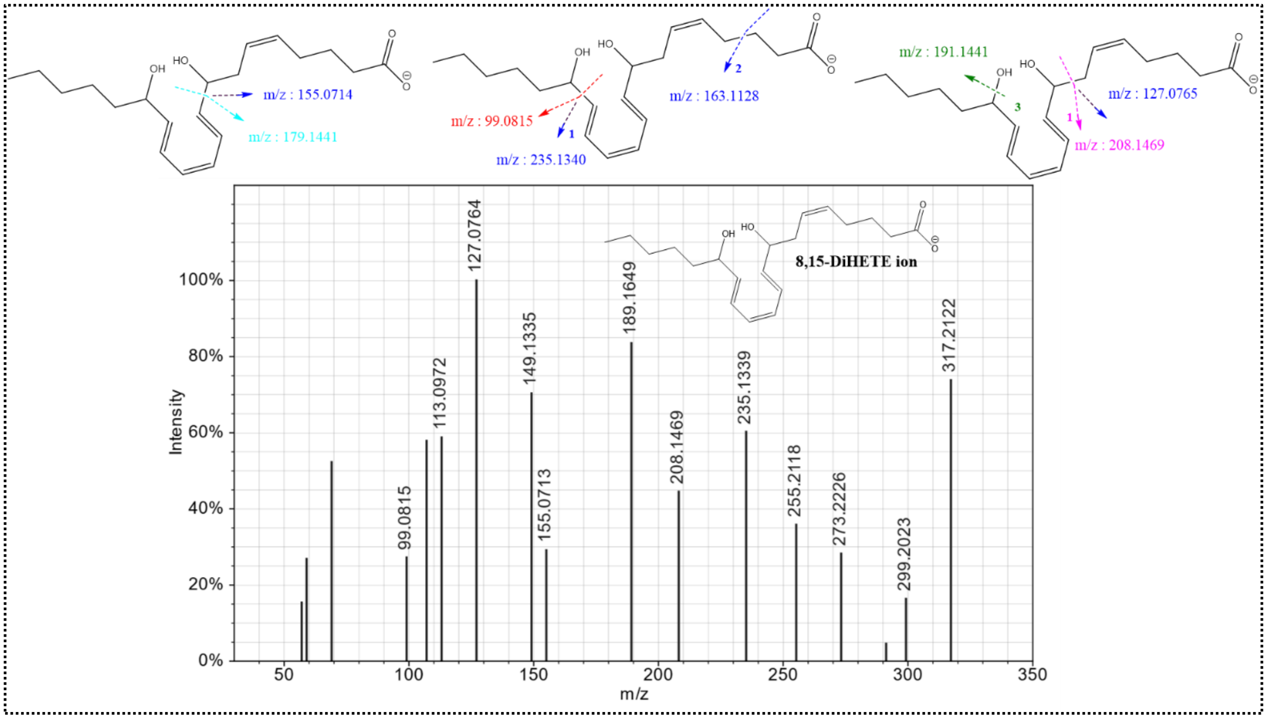

Supplementary Figure S11. **Electrospray ionization (negative ions) and tandem mass spectrometry of 14,15-DiHETE obtained from the oxidation of the 15-HETE.**

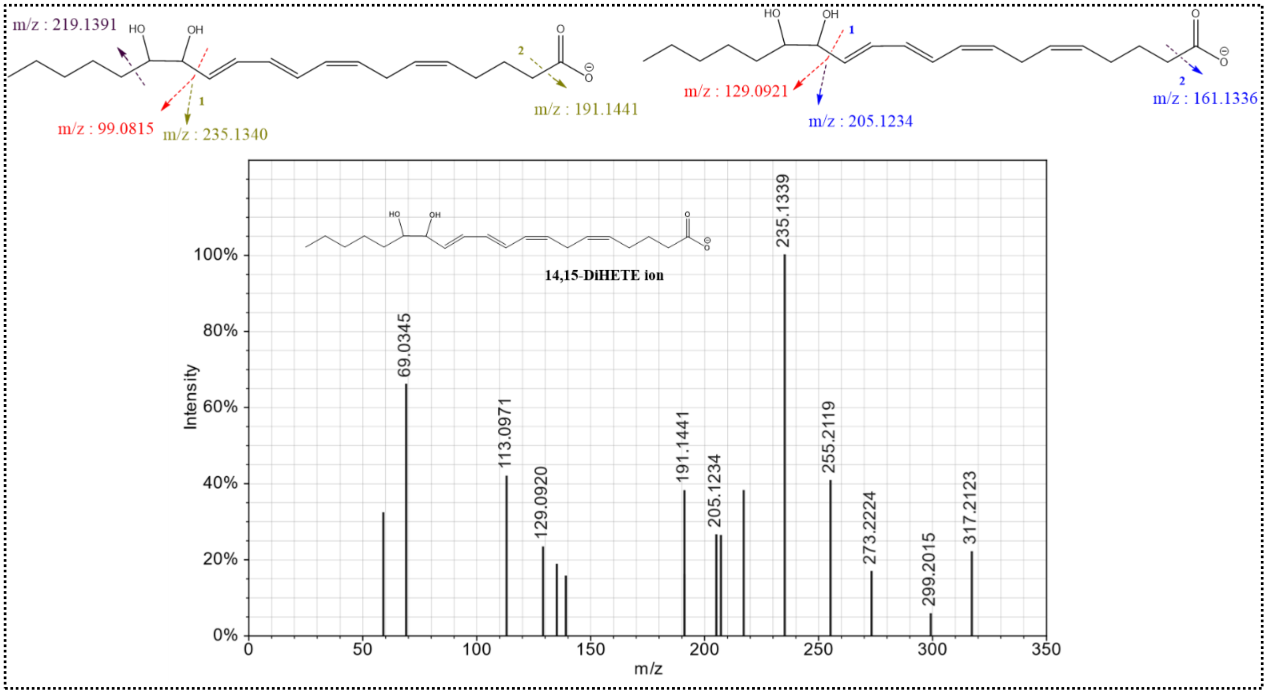

Supplementary Figure S12. Electrospray ionization (negative ions) and tandem mass spectrometry of PGH_2.

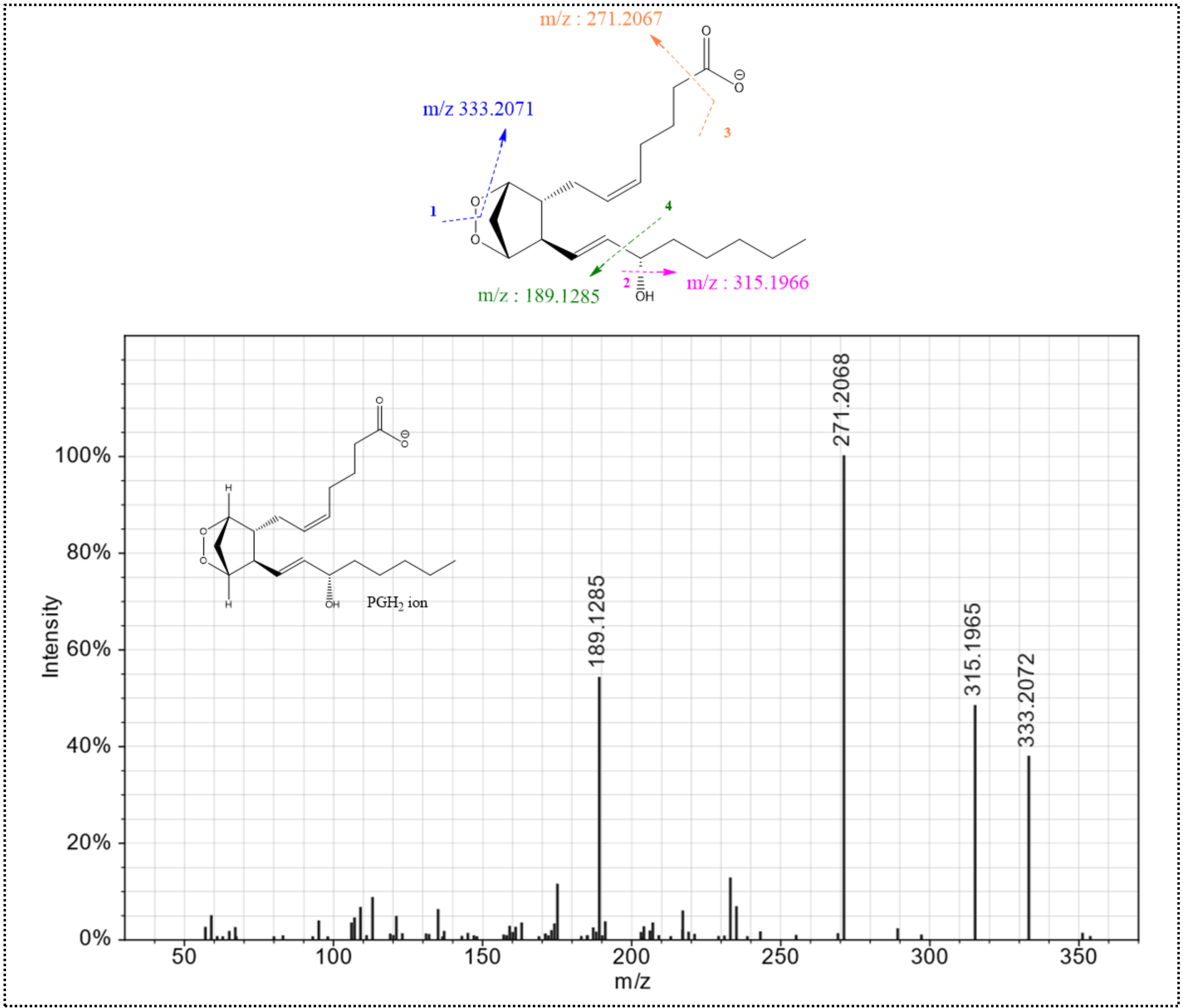

Supplementary Figure S13. **Electrospray ionization (negative ions) and tandem mass spectrometry of PGE_2 obtained from the rearrangement of the PGH_2.**

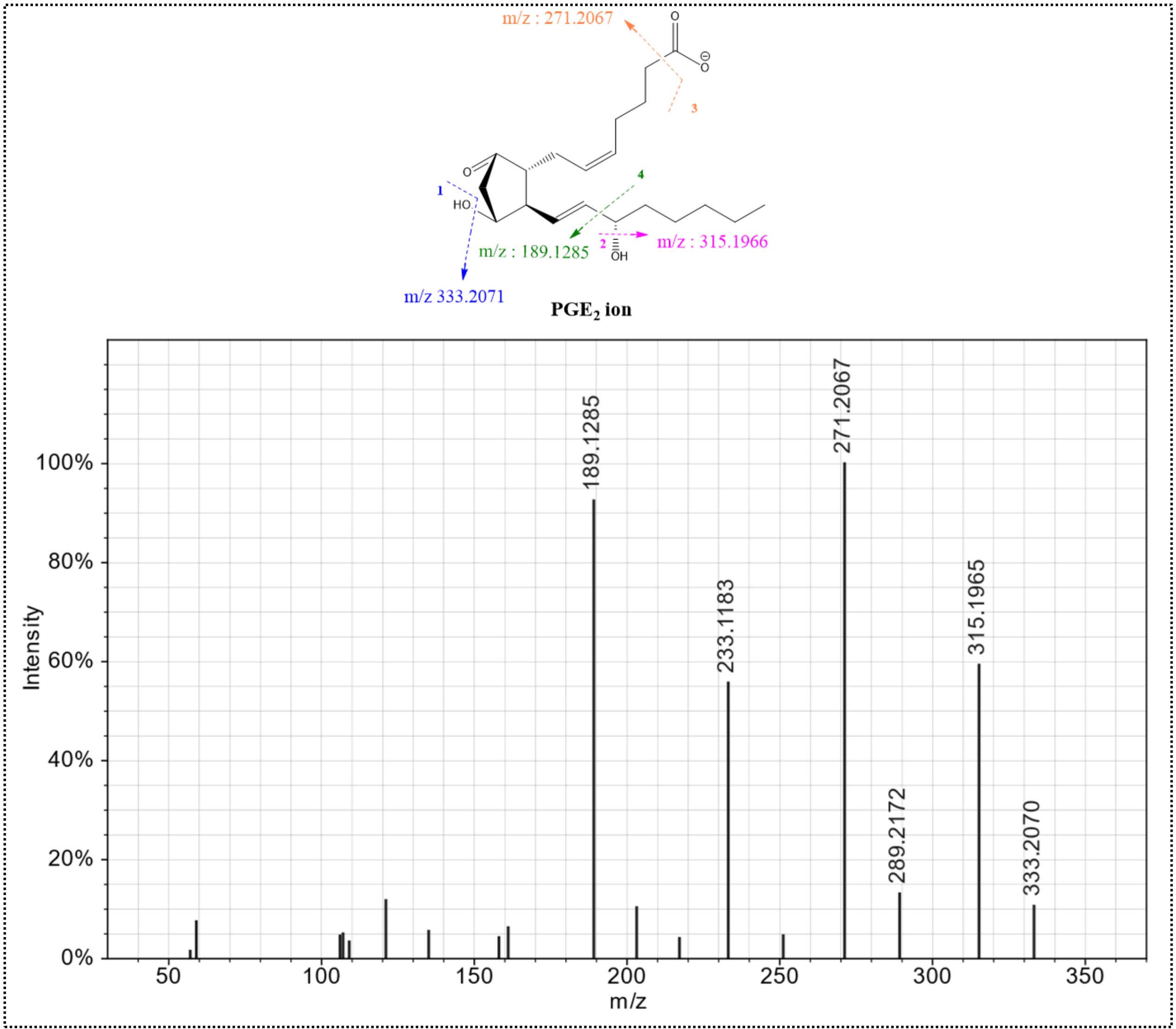

Supplementary Figure S14. Electrospray ionization (negative ions) and tandem mass spectrometry of PGD_2 obtained from the rearrangement of the PGH_2.

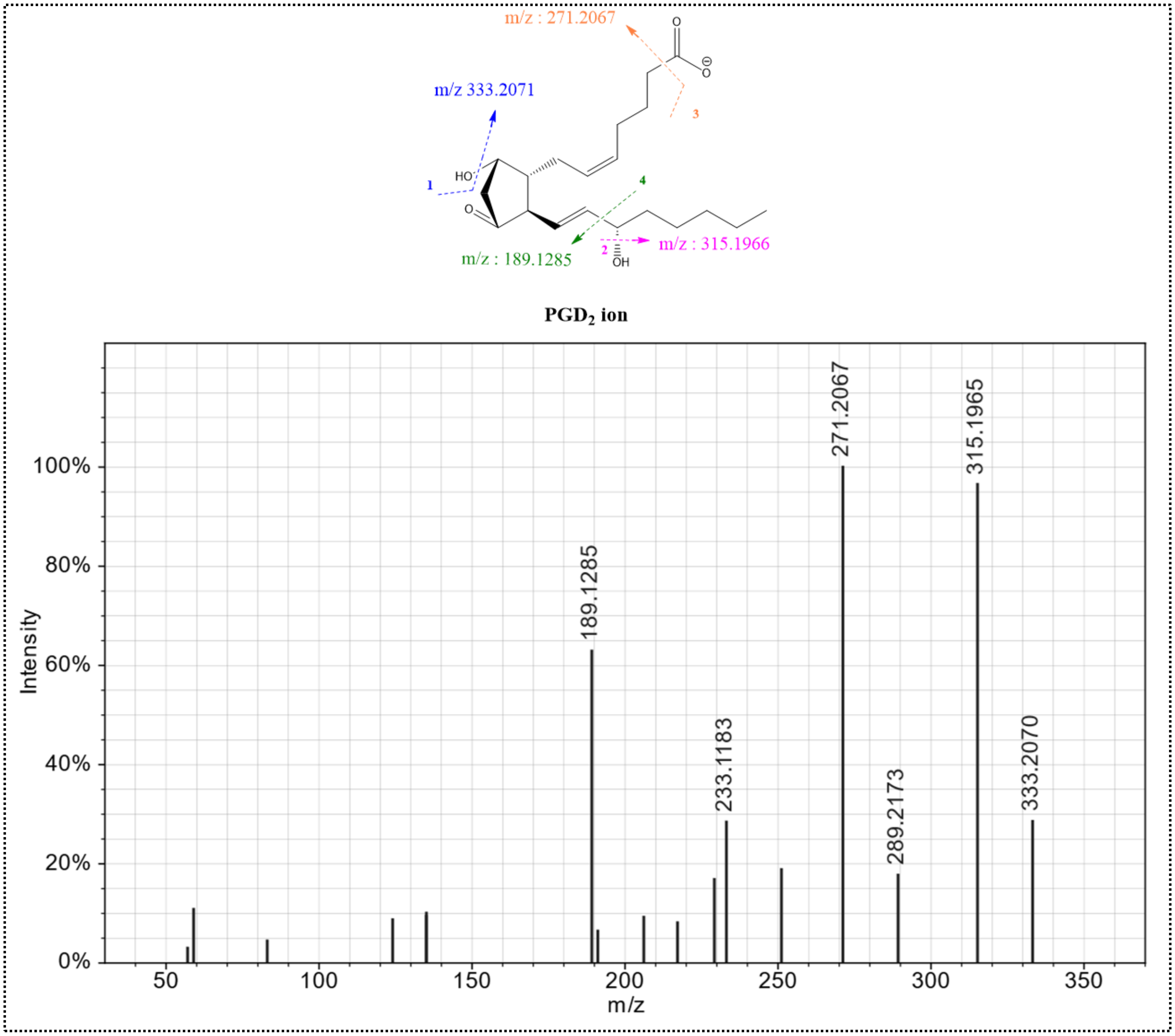

Supplementary Figure S15. Electrospray ionization (negative ions) and tandem mass spectrometry of PGF_2 obtained from the rearrangement of the PGH_2.

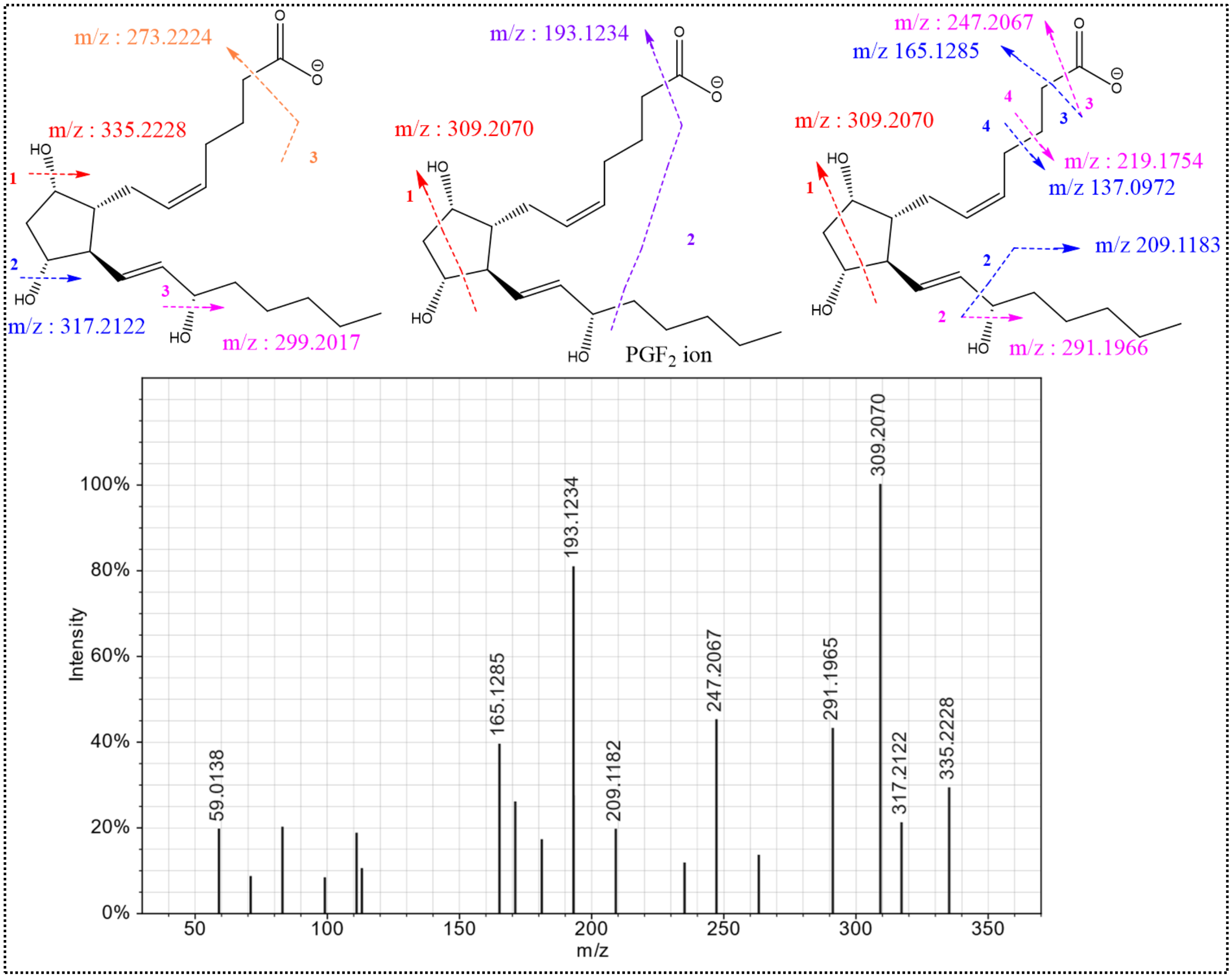

Supplementary Figure S16. **Electrospray ionization (negative ions) and tandem mass spectrometry of keto-PGE_2 obtained from the rearrangement of the PGH_2.**

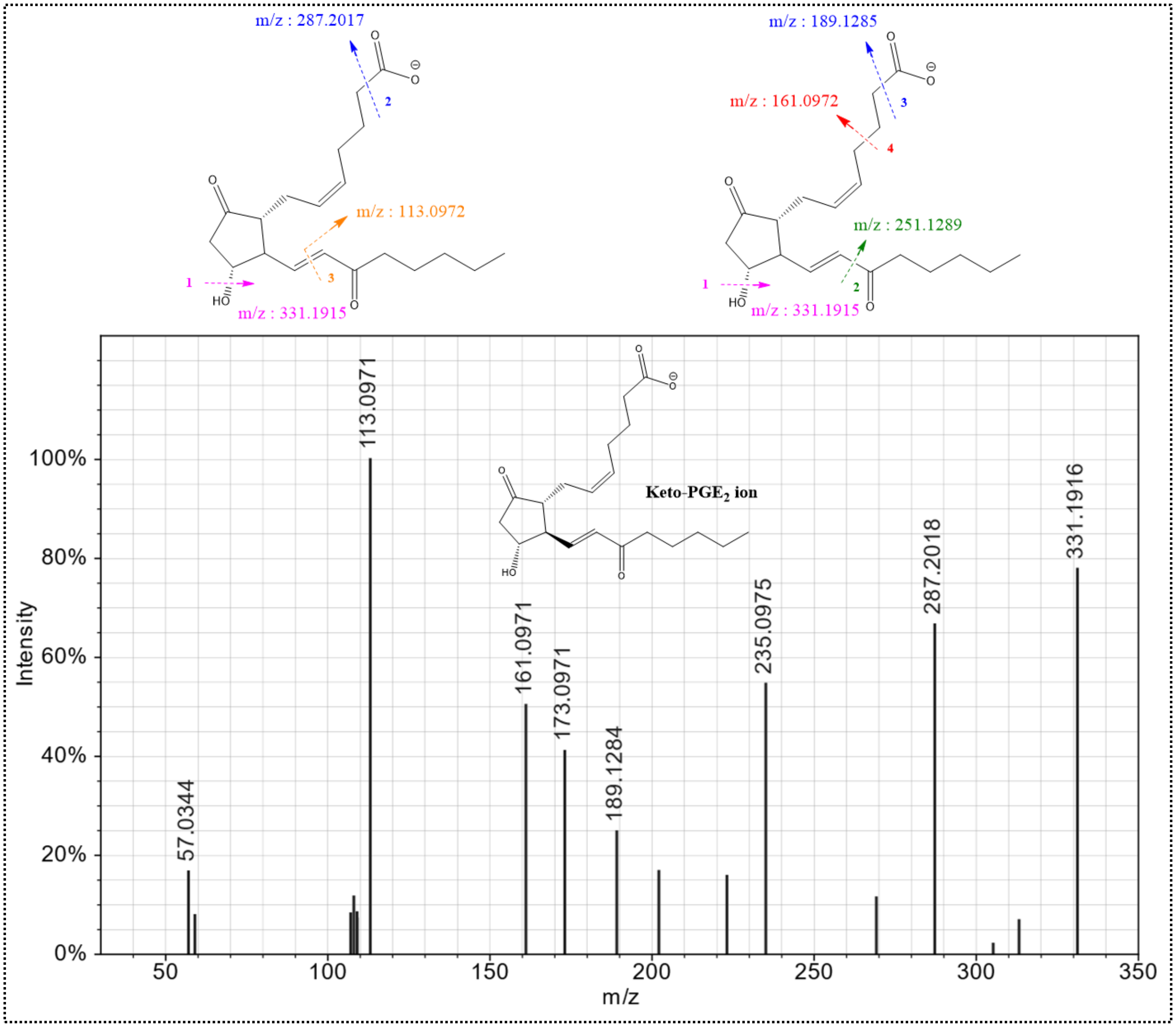

Supplementary S17. Electrospray ionization (negative ions) and tandem mass spectrometry of the compound with m/z 347.259 and the plausible specific fragments of the 13-hydroxy-7Z,10Z,14E,16Z-docosatrienoic acid (13-HDT).

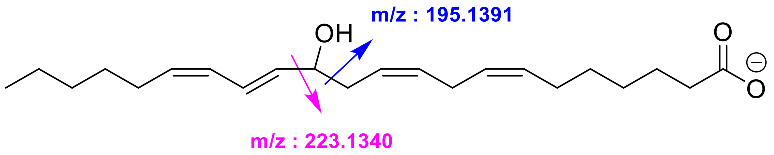

13-hydroxy-7Z,10Z,14E,16Z-docosatrienoic acid ion

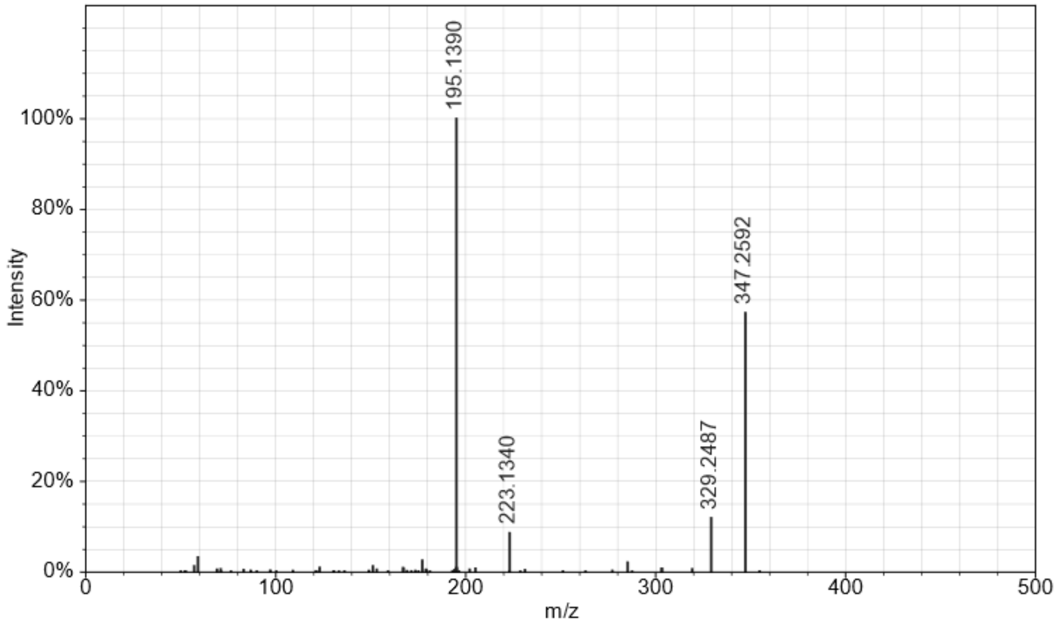

Supplementary S18. Electrospray ionization (negative ions) and tandem mass spectrometry of the compound with m/z 293.212 and the plausible specific fragments of 9-monohydroxyl of pinolenic acid : 9-hydroxy-(5Z,10Z,12Z)-octadeca-5,9,12-trienoic acid and plausible specific fragments of the 9-monohydroxyl of alpha linolenic acid : 9-hydroxy-(10E,12Z,15Z)-octadeca-9,12,15-trienoic acid.

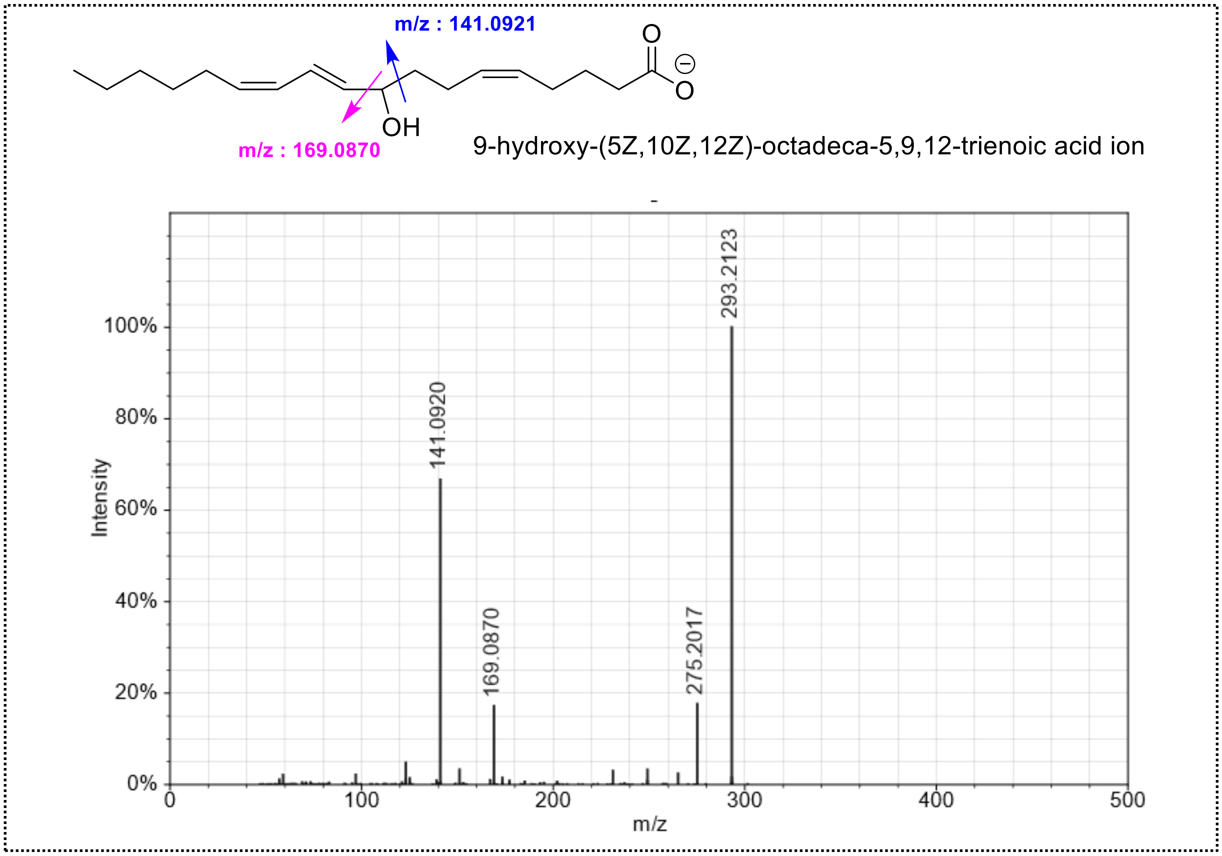

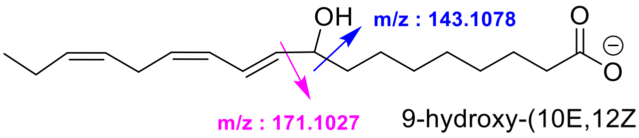

9-hydroxy-(10E,12Z,15Z)-octadeca-9,12,15-trienoic acid ion

Supplementary S19. Electrospray ionization (negative ions) and tandem mass spectrometry of the compound with m/z 345.244 and the plausible specific fragments of 13-monohydroxyl of Osbond acid : 13-hydroxy (4Z,7Z,10Z,14E,16Z)-docosapentaenoic acid.

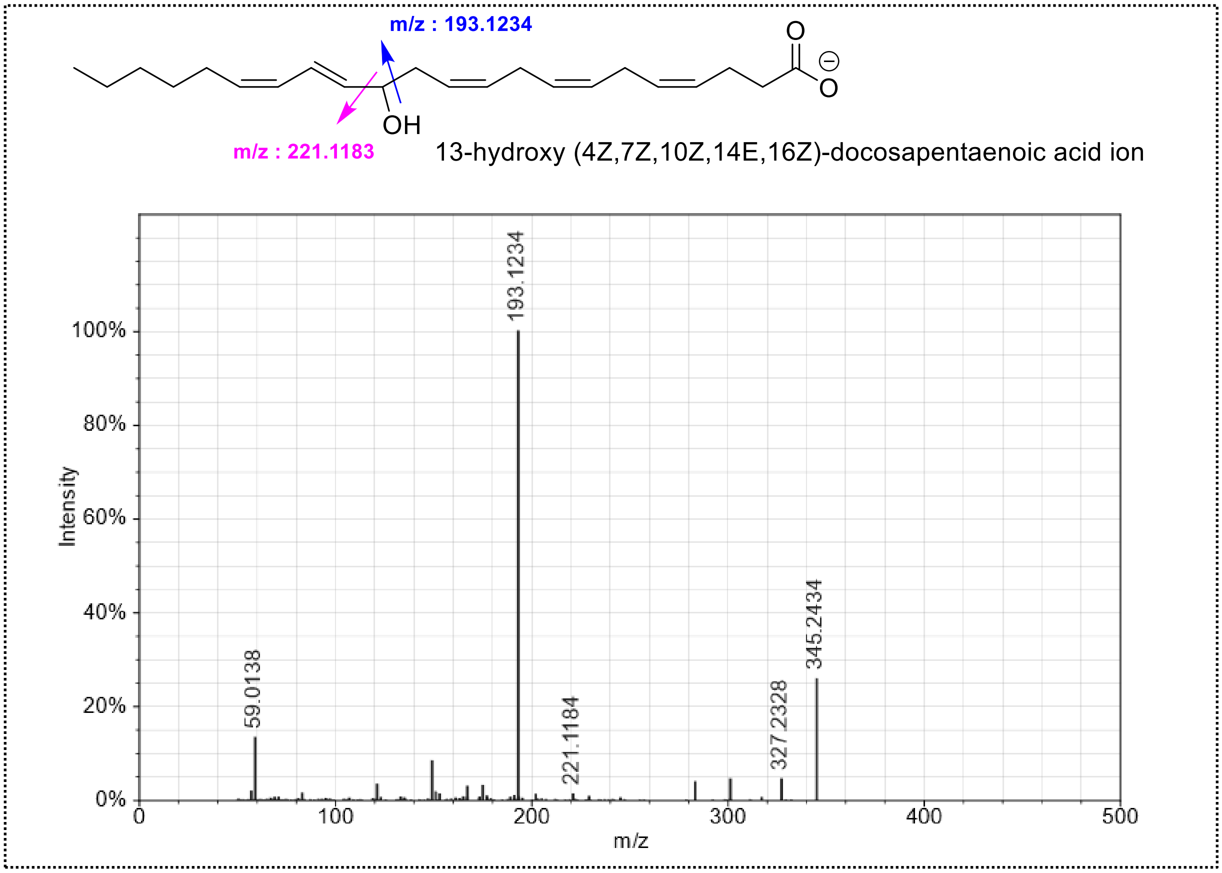

Supplementary S20. Electrospray ionization (negative ions) and tandem mass spectrometry of the compound with m/z 321.243 and the plausible specific fragments of 11-monohydroxyl series of dihomo-gamma-linolenic : 11-hydroxy-(8Z,12E,14Z)-icosa-8,11,14-trienoic acid.

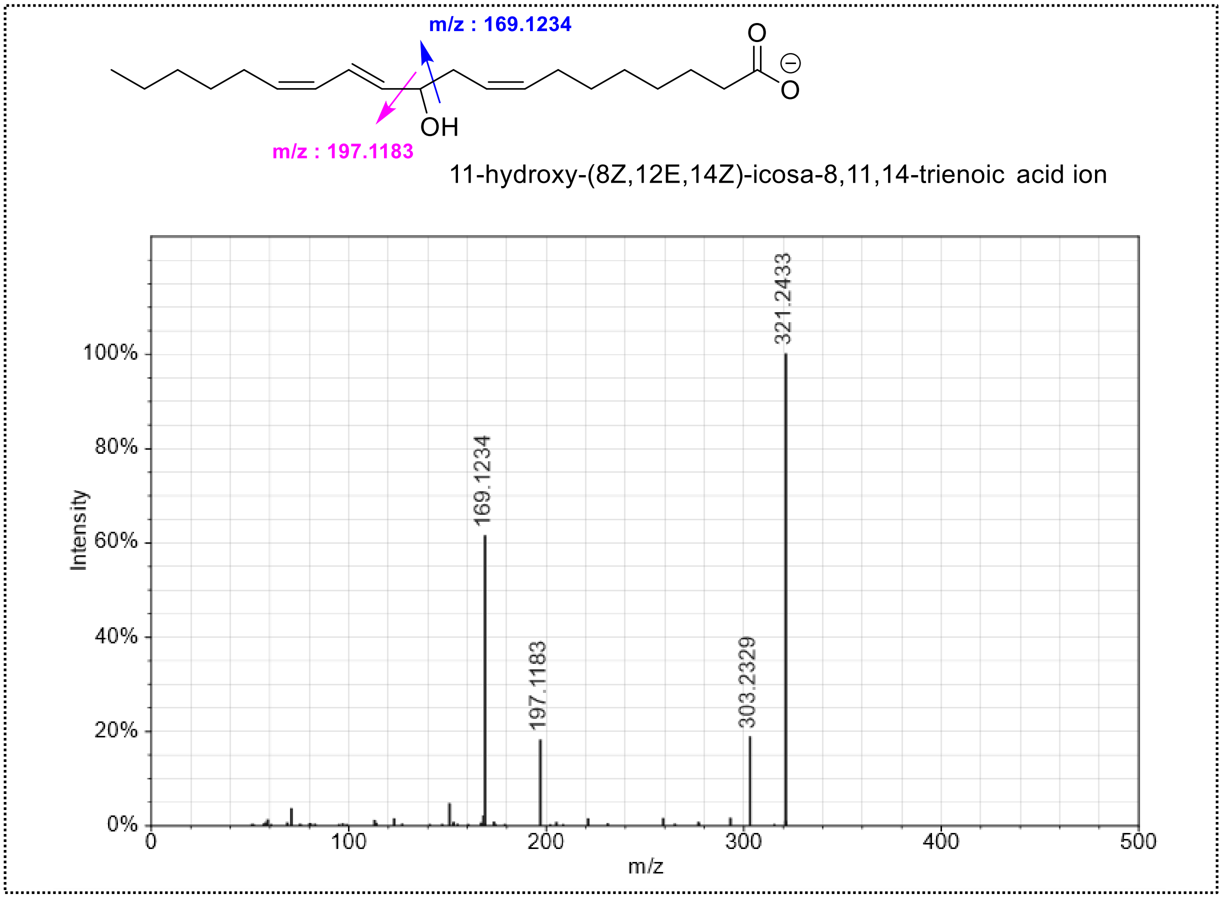

Supplementary S21. Dereplication of the NEO-MSMS against the "putative watrous' oxylipins" previously available on the GNPS library.

Six out of the eight compounds (319_22__5_31, 327_21__3_30, 327_21__4_03, 333_21__2_77, 343_22__4_31, 347_26__5_45, 347_26__5_58, and 349_24__4_63) were putatively identified based on our **NEO-MSMS** library. Three compounds were directly annotated using the MOLECULAR-LIBRARYSEARCH tool, using the following parameters: a cosine score threshold of 0.6, a minimum of 6 matched peaks, a mass tolerance of 0.1 Da, taking into account the calibration issue, and a Top Hits Per Spectrum of 50 compounds, as indicated in the table below.

Table 1 : Identification of putatives compound using MOLECULAR-LIBRARYSEARCH tool based on NEO MSMS library

Putative compound	Compound name	Cosine	GNPS Link
319_22__5_31	5_HETE__5_17	0.74	https://gnps.ucsd.edu/ProteoSAFe/status.jsp?task=ac883c3613304a14b18daea5efa7d4ad
	19_HETE	0.63	
	20_HETE	0.63	
	11_12_EET__5_51	0.62	
327_21__4_03	9-epi-9-F1t-PhytoP	0.60	https://gnps.ucsd.edu/ProteoSAFe/status.jsp?task=7841af9ed40e490eba267f2ff4d92e1a
	9-F1t-PhytoP	0.60	
343_22__4_31	20-HDHA	0.93	https://gnps.ucsd.edu/ProteoSAFe/status.jsp?task=59858b538ff942c88ddf3fd942148048
	19,20-EpDPA	0.9	
	20_HDoHE	0.81	

The compounds 343_22__4_31 and 319_22__5_31, named as oxylipin 3 and 1 in the Demler et al., 2020 study[4], were identified as 20-HDHA and 5-HETE. Despite not being directly connected in the molecular network with the corresponding "Watrous standards", they were annotated as such with those same standards (now introduced in **NEO-MSMS**) using the "Library search" tool of GNPS. This is likely due to the fact that the network connections consider the maximum number of neighbor nodes for one single node and the maximum size of nodes allowed in a single connected network.

Explanations: Since, none of the 134 detected oxylipins in the study were reported (being the only data missing in the report, *"the commercial pool plasma dereplication sample"),* we assumed that this identified putative 5-HETE is another isomer of the enzymatic 5(S)-hydroxy-6E,8Z,11Z,14Z-eicosatetraenoic acid having a retention time difference (5.17 min vs 5.31 min). Hence, we came to the conclusion that it could be the non-enzymatically produced NEO-PUFA 5(RS)-hydroxy-6E,8E,11Z,14Z-eicosatetraenoic typically formed during reversible addition of oxygen to pentadienyl radical intermediates.[5] It has to be noted that the spectra is poor in fragments, and care should be taken for this elucidation.

Similarly, we believed that 343_22__4_31 is the enzymatic isomer of 20-HDHA having a retention time difference (4min31 vs 4.48min). This time, the spectra is richer and the cosine of high value.

As we can observe in those 2 examples, despite a significant similarity in fragmentation between the unknown and reference compounds, the decimal error on the fragments is considerable due to the lack of MS2 accurate calibration for the unknown compounds derived from the study by Watrous et al 2019[6]. Like explained it prompted us to work with an error tolerance of 0.1 Da on the parent ion and fragments.

Below, the two figures show the mirror spectra of the references with the putative compounds.

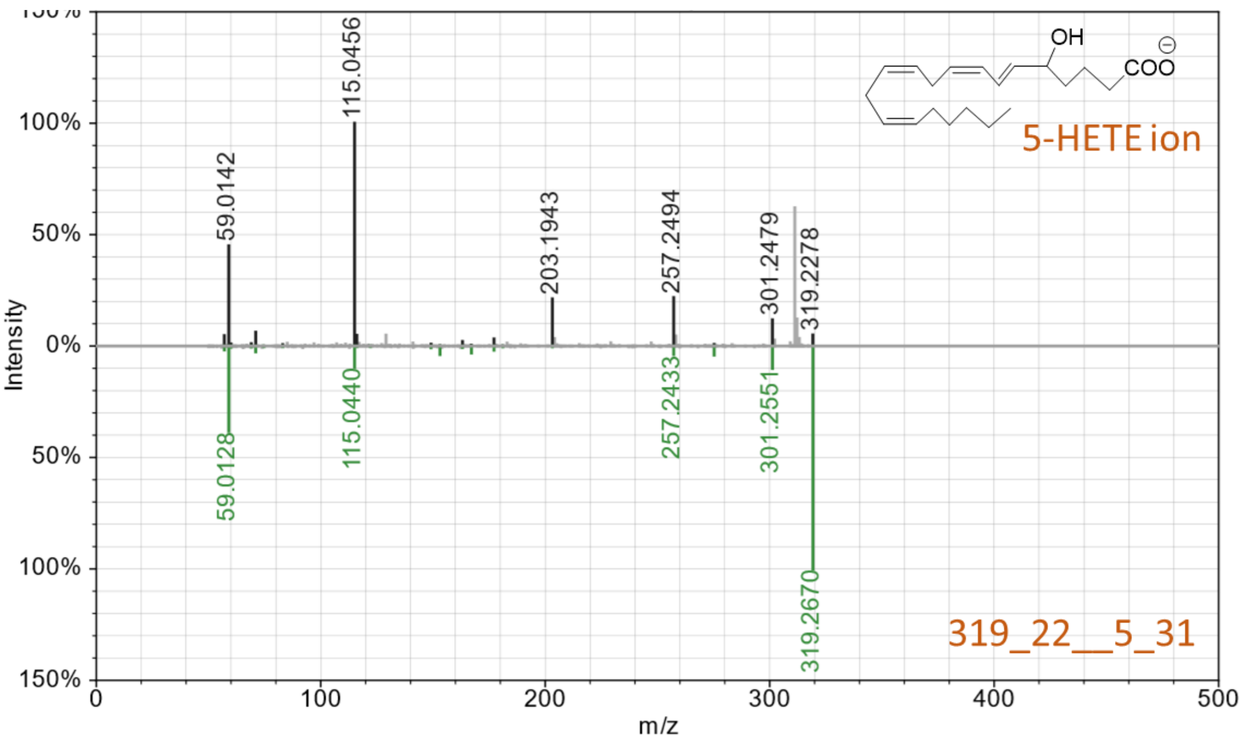

Figure 1 : MS/MS spectra mirror of the unknown 319_21__5_31 compound with 5-HETE reference

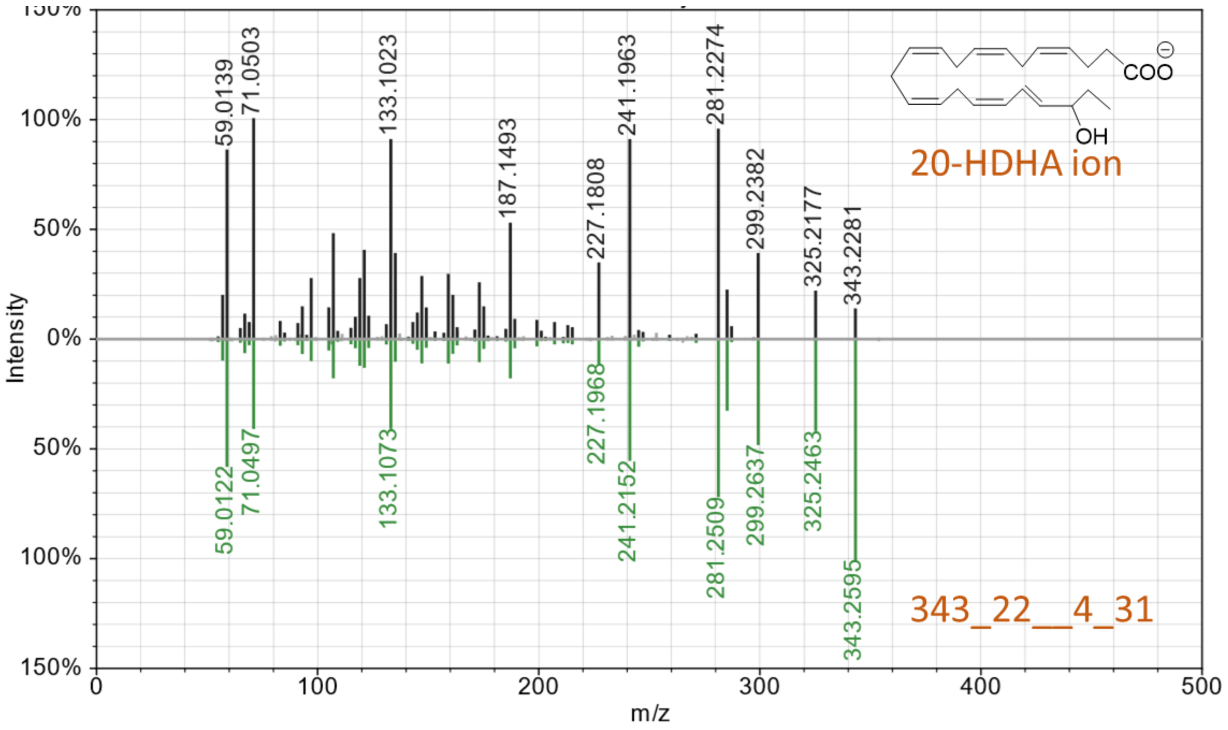

Figure 2 : MS/MS spectra mirror of the unknown 343_22__4_31 compound with 20-HDHA reference

The third compound, 327_21__4_03, was annotated as 9-epi-9-F_{1t}-PhytoP. However, due to the paucity of the number of fragments and the off calibration, great care should be taken here again. That characteristic fragment at the 9th position (m/z 171) is certainly indicative of the position of the allylic alcohol, but the characteristic ethanal loss of isoprostanoid (to not be confounded with CO_2 loss, see figure S1) cannot be retrieved due to the poor calibration. However, $C_{18}H_{32}O_5$ formula can also correspond to triol derivatives of C18 PUFAs, but a characteristic fragment at 171 are not trivial to explain for any triols we can think of, but is always a possibility. To the concern of 9-epi-9-F_{1t}-PhytoP being already detected in plasma sample is however very recurrent in the NEO-PUFA quantitation in human samples[7,8] so we could easily make sense of its presence.

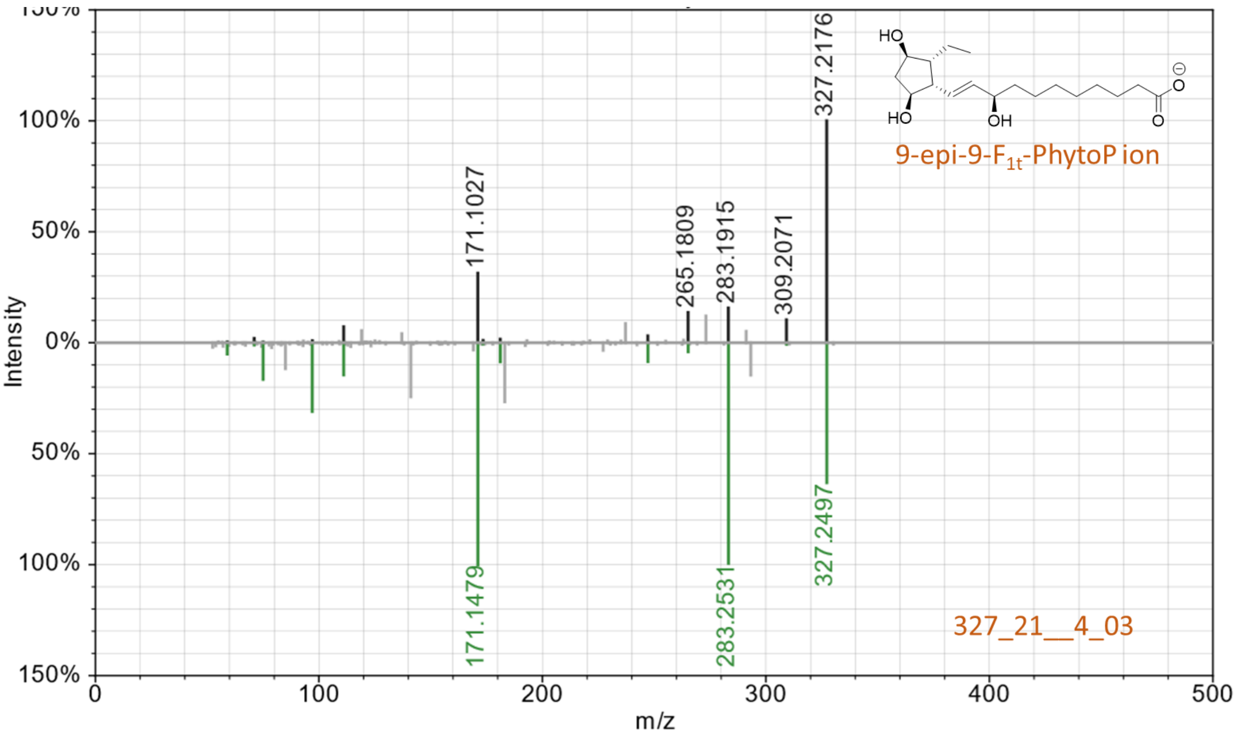

Figure 3 : MS/MS spectra mirror of the unknown 327_21__4_03 compound with 9-epi-9-F_{1t}-PhytoP reference

Subsequently, through a reanalysis of the molecular network data presented by Watrous et al. in 2019, utilizing the same parameters and incorporating our library, three analogous structures were proposed for three of the remaining five unidentified compounds: 333_21__2_77, 347_26__5_58, and 347_26__5_45.

Putative compounds	Analogue	Masse difference
333_21__2_77	14(S)-HDHA	10 Da
347_26__5_58	11-HEPE	30 Da
347_26__5_45	Massbank:IA000425 11-HETE	28 Da

The subsequent analysis revealed that two compounds, 347_26__5_58 and 347_26__5_45, showed an increased mass of +30 and +28 respectively with library matching 11-HEPE and 11-HETE. It is probably an clear indication that these two putative unknowns are monohydroxyl derivatives of adrenic acid with the formula $C_{22}H_{36}O_3$ (m/z 347). Compound 347_26__5_58 was found to correspond perfectly to 11-hydroxy-7E,9Z,13Z,16Z-docosatrienoic acid (11-HDT) based on the analysis of MSMS fragments. *It has to be noted that we assigned the conjugate double-bonds as E,Z, but its NEO-PUFA version could likely be E,E.*

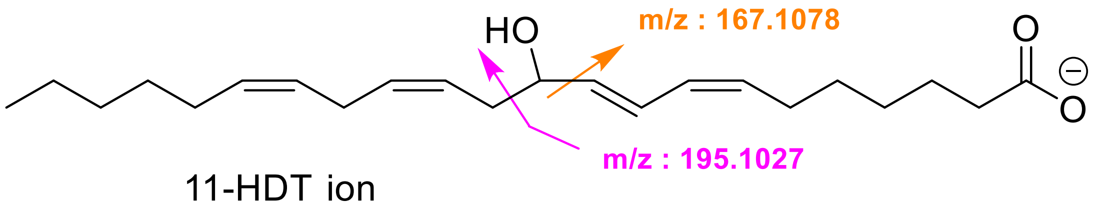

Figure 4 : plausible Specific fragment of 11-hydroxy-7E,9Z,13Z,16Z-docosatrienoic acid ((11-HDT)

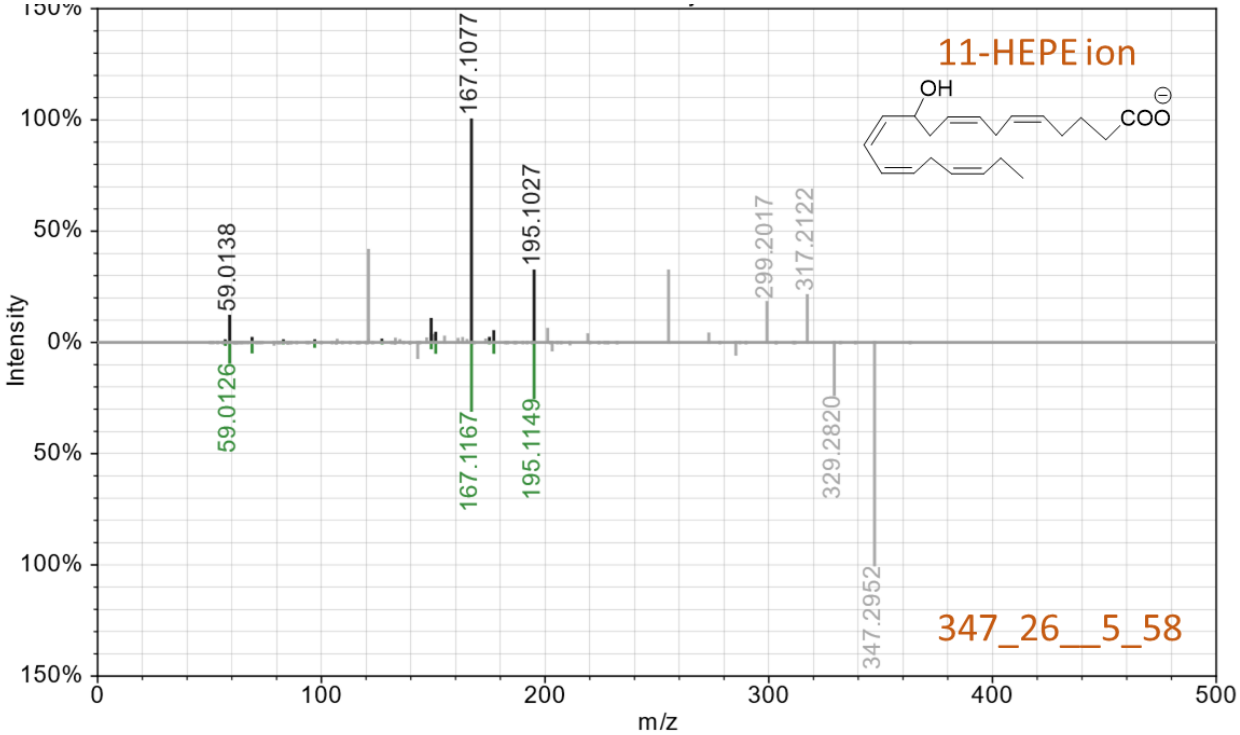

Figure 5 : MS/MS spectra mirror of the unknown 347_26__5_58 compound with 11-HEPE reference

Regarding the second compound, 347_26__5_45, the position of the OH group in relation to the chain length plays a crucial role in connecting PUFA derivatives. In the case of 11-HETE from a 20-carbon AA (arachidonic acid), it tends to be more closely associated with the C22 series. The observed fragments of 13-hydroxy-7E,10E,14E,16Z-docosatrienoic acid (13-HDT) align well with those present in the spectrum of the putative compound. It has to be noted that we assigned the conjugate double-bonds *as E,Z,* but its NEO-PUFA version could likely be *E,E.*

It is important to note that these fragments were employed in the identification process based solely on the integer values of their mass, without considering the decimal part. However, because being the simplest formulae possible of oxylipins (NEO-PUFA) it makes little doubt as being the annotated compounds described herein.

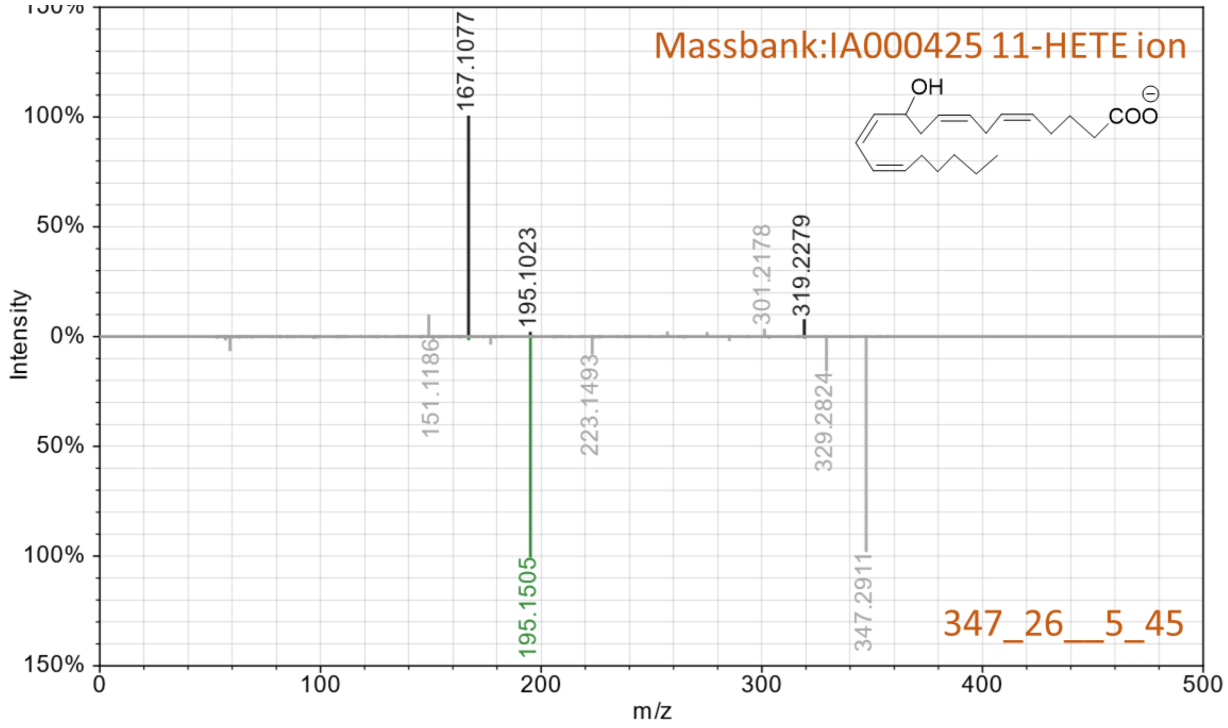

Figure 6 : MS/MS spectra mirror of the unknown 347_26__5_45 compound with 11-HETE reference

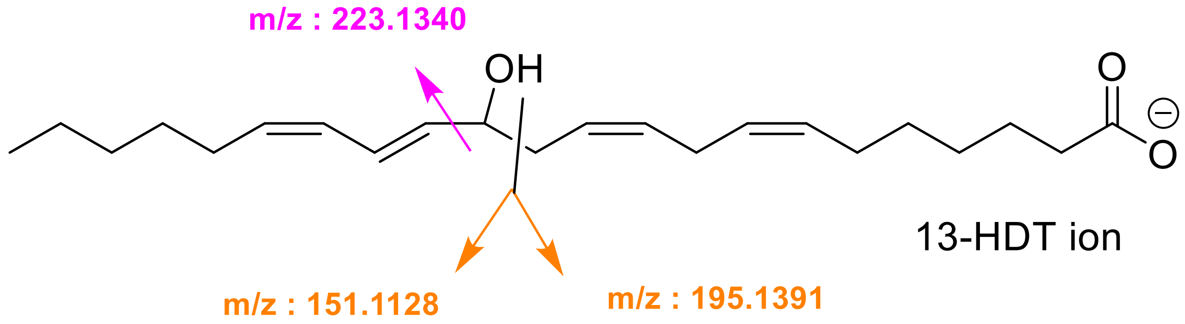

Figure 7 : plausible specific fragment of 13-HDT

Regarding the last compound (333_21__2_77) which mirrored with 14(S)-HDHA from **NEO-MSMS**, the initial losses are identical in both compounds, involving H_2O and then CO_2, maintaining a mass difference of 10 Da between the parent ions as well as between the first fragments (m/z 325 and m/z 281 for 14-HDHA, and m/z 315 and m/z 371 for the unknown compound).

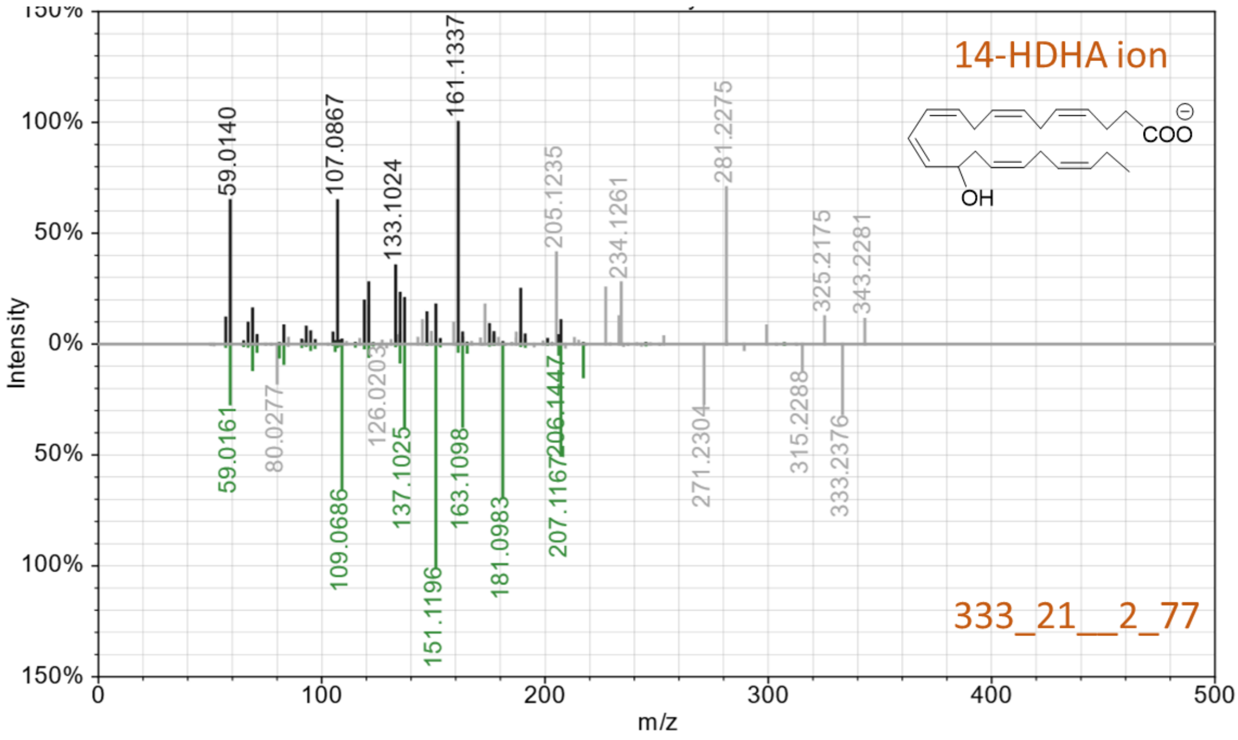

Figure 8 : MS/MS spectra mirror of the unknown 333_21__2_77 compound with 14(S)-HDHA reference

Furthermore, from the major fragments of known 14-HDHA, we observed a 2 Da difference in three significant fragments (m/z 205, 161, and 107) compared to the spectrum of the unknown compound. These three fragments result from successive fragmentations, as illustrated in the following figure.

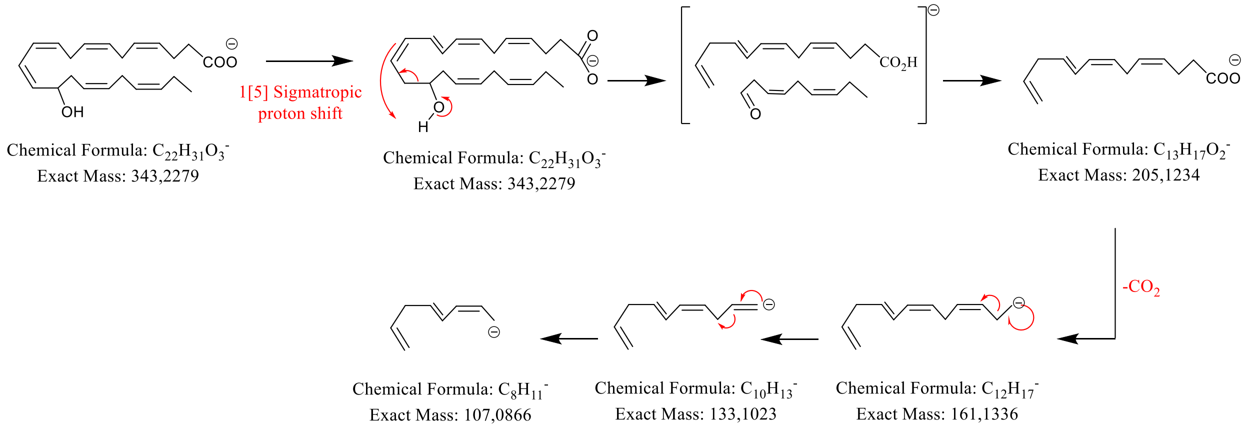

Figure 9 : Successive fragmentations of deprotomer 14-HDHA in negative mode

It is likely that the unknown compound is a derivative sharing the same end as 14-HDHA, hence possibly a derivative of AA or EPA. The two ways to obtain an m/z 207 fragment, which can then lose a CO_2 to yield m/z 163, are to have the OH group at positions 12 or 14, as indicated in the figure 10. Thanks to the non-calibrated MS2 we can't tell them apart in the spectra. The other incremental 2 Da fragments could be explained with those two possible fragments.

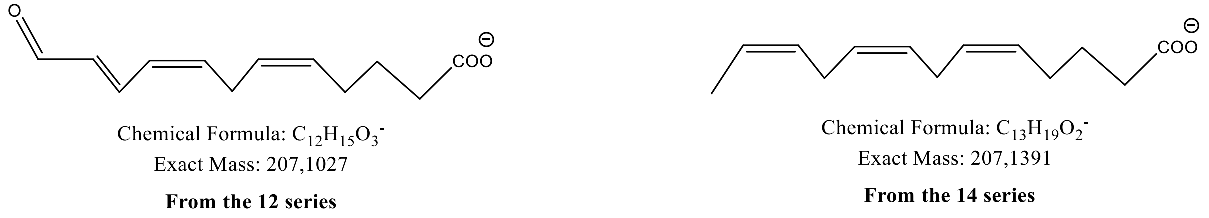

Figure 10 : Possible m/z 207 fragments based on a 14-HDHA skeleton with a precursor ion of C20 formulae

It is also important to note that 14-HDHA ESI(-) fragmentation yields the even mass-to-charge ratio-(m/z 234) fragment known to occur via oxy-Cope rearrangement and homolytic cleavage.

Interestingly, our unknown compound also possesses event masses m/z 206 and 126. Those can only be explained with the 14,17-DiHEPE metabolite, a NEO-PUFA from EPA (as far as we know no enzyme can make it). Those even masses can be obtained by oxy-Cope rearrangement of deprotonated 14,17-DiHEPE followed by homolytic cleavage of the corresponding ion dipole (See Figure below).

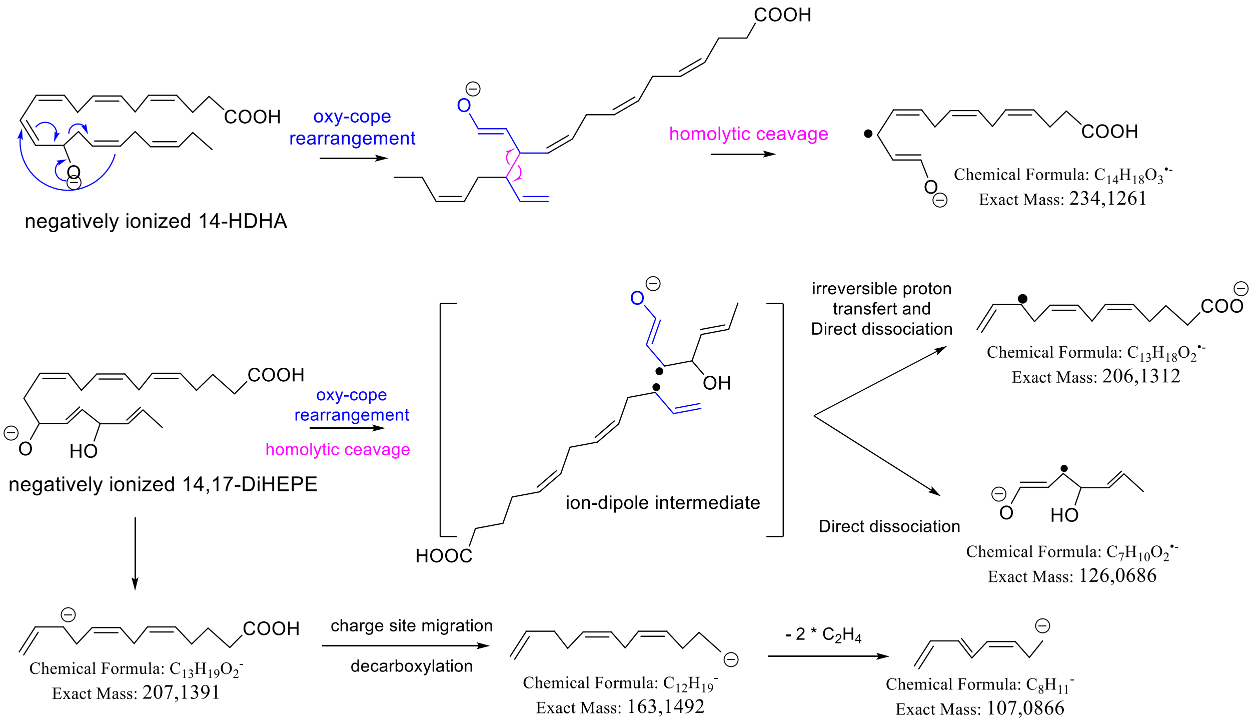

Figure 11 : Some characteristic fragments explained through the oxy cope rearrangement for 14-HDHA and putative 14,17-DiHEPE

References

1. Pinet, A., Nguyen, T. L., Bernadat, G., Figadère, B. & Ferrié, L. Pinet, A.; Nguyen, T. L.; Bernadat, G.; Figadère, B.; Ferrié, L. Synthesis of 3,5-Disubstituted 1,2-Dioxolanes through the Use of Acetoxy Peroxyacetals. Org. Lett. 2019, 21 (12), 4729–4733. https://doi.org/10.1021/acs.orglett.9b01616. *Org. Lett.* **21**, 4729–4733 (2019).
2. Nguyen, T., Ferrié, L. & Figadere, B. Nguyen, T. L.; Ferrié, L.; Figadère, B. Synthesis of 3,5-Disubstituted-1,2-Dioxolanes: Access to Analogues of Mycangimycin and Some Rearrangement Products. Tetrahedron Letters 2016, 57 (47), 5286–5289. https://doi.org/10.1016/j.tetlet.2016.10.051. *Tetrahedron Lett.* **57**, (2016).
3. Hong, S. *et al.* Resolvin D1, protectin D1, and related docosahexaenoic acid-derived products: Analysis via electrospray/low energy tandem mass spectrometry based on spectra and fragmentation mechanisms. *J. Am. Soc. Mass Spectrom.* **18**, 128–144 (2007).
4. Demler, O. V. *et al.* One-Year Effects of Omega-3 Treatment on Fatty Acids, Oxylipins, and Related Bioactive Lipids and Their Associations with Clinical Lipid and Inflammatory Biomarkers: Findings from a Substudy of the Vitamin D and Omega-3 Trial (VITAL). *Metabolites* **10**, 431 (2020).
5. Punta, C., Rector, C. L. & Porter, N. A. Peroxidation of polyunsaturated fatty acid methyl esters catalyzed by N-methyl benzohydroxamic acid: a new and convenient method for selective synthesis of hydroperoxides and alcohols. *Chem. Res. Toxicol.* **18**, 349–356 (2005).
6. Watrous, J. D. *et al.* Directed Non-targeted Mass Spectrometry and Chemical Networking for Discovery of Eicosanoids and Related Oxylipins. *Cell Chem. Biol.* **26**, 433-442.e4 (2019).
7. Galano, J.-M. *et al.* Isoprostanes, neuroprostanes and phytoprostanes: An overview of 25 years of research in chemistry and biology. *Prog. Lipid Res.* **68**, 83–108 (2017).
8. Barden, A. E. *et al.* Flaxseed Oil Supplementation Increases Plasma F1-Phytoprostanes in Healthy Men1,2. *J. Nutr.* **139**, 1890–1895 (2009).
